# Qudit-based variational quantum eigensolver using photonic orbital angular momentum states

**DOI:** 10.1126/sciadv.ado3472

**Published:** 2024-10-23

**Authors:** Byungjoo Kim, Kang-Min Hu, Myung-Hyun Sohn, Yosep Kim, Yong-Su Kim, Seung-Woo Lee, Hyang-Tag Lim

**Affiliations:** ^1^ Center for Quantum Technology, Korea Institute of Science and Technology (KIST), Seoul 02792, Korea.; ^2^ Department of Laser & Electron Beam Technologies, Korea Institute of Machinery and Materials (KIMM), Daejeon 34103, Korea.; ^3^ Division of Quantum Information, KIST School, Korea University of Science and Technology, Seoul 02792, Korea.; ^4^ Department of Applied Physics, Kyung Hee University, Yongin 17104, Korea.; ^5^ Department of Physics, Korea University, Seoul 02841, Korea.

## Abstract

Solving the electronic structure problem is a notorious challenge in quantum chemistry and material science. Variational quantum eigensolver (VQE) is a promising hybrid classical-quantum algorithm for finding the lowest-energy configuration of a molecular system. However, it typically requires many qubits and quantum gates with substantial quantum circuit depth to accurately represent the electronic wave function of complex structures. Here, we propose an alternative approach to solve the electronic structure problem using VQE with a single qudit. Our approach exploits a high-dimensional orbital angular momentum state of a heralded single photon and notably reduces the required quantum resources compared to conventional multi-qubit–based VQE. We experimentally demonstrate that our single-qudit–based VQE can efficiently estimate the ground state energy of hydrogen (H_2_) and lithium hydride (LiH) molecular systems corresponding to two- and four-qubit systems, respectively. We believe that our scheme opens a pathway to perform a large-scale quantum simulation for solving more complex problems in quantum chemistry and material science.

## Teaser

A single photon’s orbital angular momentum qudit state can carry out quantum chemistry calculations.

## INTRODUCTION

With the advent of quantum computing, there has been growing interest in developing efficient variational quantum algorithms (VQA) capable of simulating molecular systems on quantum devices (*1*). Among them, the variational quantum eigensolver (VQE) is a promising candidate to realize near-term quantum computers with quantum advantages in the noisy intermediate-scale quantum (NISQ) era (*2*, *3*). VQE is well-known as a hybrid classical-quantum algorithm for finding the ground state energy of a many-body interacting fermionic Hamiltonian that combines a quantum subroutine with a classical optimization loop. The VQE algorithm involves varying the parameters of a trial wave function, called the ansatz state, to minimize the expectation value of the Hamiltonian. This optimization process is repeated iteratively until the convergence of the ground state energy is achieved (*4*). The VQE has been implemented as multi-qubit systems in various quantum hardware architectures including photonic (*4*, *5*), superconducting (*6*–*9*), and trapped-ion qubit systems (*10*). However, to estimate the ground state energy of the electronic structure of more complex molecules with VQE, quantum circuits containing many qubits and quantum gates are necessary, which is still challenging in most of physical platforms (*4*, *7*–*10*).

An alternative approach to increase the computing power of VQE is using a high-dimensional quantum state, i.e., qudit. A qudit corresponds to a *d*-dimensional system and has several advantages in quantum computing, such as reduction of quantum resources and noise resilience (*11*–*13*). Accordingly, the qudit systems have been actively investigated across various platforms including superconducting (*14*, *15*) and trapped-ion systems (*16*, *17*). However, they encounter the challenges in dimensional scalability due to increased gate implementation complexity and reduced gate fidelity (*14*–*17*). The orbital angular momentum (OAM) of a single photon is another attractive option to realize high-dimensional quantum states (*18*–*21*). Because it is possible to prepare, manipulate, and measure OAM states of a single photon with hundreds of Laguerre Gaussian (LG) modes, various quantum information processing using OAM qudit states has been reported in the fields of quantum computing (*22*), quantum cryptography (*23*), and quantum metrology (*24*). However, despite its potential advantages, practical applications using OAM qudit states in quantum computation and simulation have been limited so far.

In this work, we experimentally demonstrate a single qudit–based variational quantum eigensolver (SQD-VQE) using OAM states of a single photon. Our SQD-VQE estimates the ground state of the four-dimensional hydrogen (H_2_) and the 16-dimensional lithium hydride (LiH) molecules, and our experimental results show that a high-dimensional OAM qudit provides high computational precision and speed in VQE by replacing multiple qubit gate operations with a single qudit gate operation based on a spatial light modulator (SLM). It markedly reduces the number of quantum registers, quantum gates, and measurements compared to the conventional qubit-based VQE. We emphasize that our proposed SQD-VQE is one of the most resource-efficient quantum processor structures for estimating the ground state energy of molecules with high performance.

## RESULTS

To construct the target Hamiltonian operator, we start from the ab initio molecular Hamiltonian according to the Born-Oppenheimer approximation (*25*). Its second quantization Hamiltonian can be expressed with fermionic operators such as creation ($\hat{a}$) and annihilation (${\hat{a}}^{†}$) operators. Furthermore, by performing the Jordan-Wigner transformation, we can represent the Hamiltonian as the Pauli strings with weight coefficients (*26*). The electronic structure of the molecular system is precisely calculated by the Hamiltonian operator with the lowest eigenvalue of *H* corresponding to the eigenvector ∣ψ〉. To estimate the lowest energy *E*_0_ at a desired interatomic distance, VQE minimizes the Rayleigh quotient based on the variational principle

$$R\left(H,|\psi \rangle \right)=\frac{\langle \psi |H|\psi \rangle }{\langle \psi |\psi \rangle }\geq E_{0}$$ (1)

where the denominator of the Rayleigh quotient can be ignored if we consider the normalized eigenvalue such that 〈ψ∣ψ〉 = 1. The numerator of the Rayleigh quotient 〈ψ∣*H*∣ψ〉 = 〈*H*〉 is the expectation value of the Hamiltonian. In VQE, the expectation value is measured by the Hamiltonian consisting of the Pauli string. By using the Jordan-Wigner transformation, any multi-qubit Hamiltonian can be decomposed into a tensor product of Pauli operators with weight coefficients as follows

$$H=\sum_{m}W_{m}P_{m}$$ (2)

where *W*_m_ are weight coefficients in real numbers, and *P*_m_ ∈ {*I*, *X*, *Y*, *Z*}^⊗*N*^ is the tensor product of multiple Pauli operators. Thus the expectation value of Hamiltonian can be obtained as a linear combination of the Pauli string expectation 〈*P*_m_〉 with weights *W*_m_ as the following

$$\langle H\rangle =\sum_{m}W_{m}\langle P_{m}\rangle$$ (3)

In VQE, we set an ansatz state at *n*th iteration $|\psi \left(\alpha_{n}\right)\rangle$ with parameterized quantum operations and read its Hamiltonian expectation value. At each iterations, using a classical optimizer, new parameters $\alpha_{n+1}$ are updated to minimize the expectation value. This minimization process is repeated until the energy expectation value converges, and the value is considered as the ground state energy.

In SQD-VQE, an ansatz state is described as a vector in a *d*-dimensional Hilbert space ℋ*_d_* spanned by the orthonormal basis ∣0〉, ∣1〉, ⋯, ∣*d* − 1〉, i.e., $|\psi \rangle =\sum_{i=0}^{d-1}c_{i}|i\rangle$, where *c_i_* are probability amplitudes with complex numbers satisfying $\sum_{i=0}^{d-1}{|c_{i}|}^{2}=1$. As the dimension *d* of qudit increases, the amount of the information can be encoded in a single qudit state increases, meaning that we are capable of performing the VQE for more complex molecules.

**Fig. 1.**
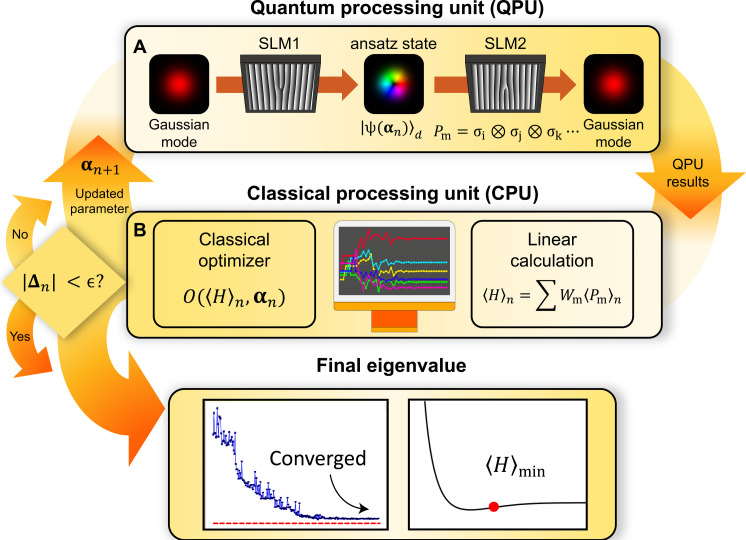
OAM qudit-based VQE procedure. (**A**) An ansatz state $|\psi \left(\alpha_{n}\right)\rangle$ is prepared and a set of Pauli projective measurements for *P*_m_ are performed using a pair of SLMs on the QPU. (**B**) CPU estimates the energy by mapping the results of the QPU with the weight coefficients. Afterward, a new set of parameters $\alpha_{n+1}$ is updated by the classical optimizer to iteratively estimate the ground state of the Hamiltonian *H*. The iteration is repeated until the squared change of the parameters ($|\Delta_{n}|$, where $\Delta_{n}\equiv \alpha_{n+1}-\alpha_{n}$) is smaller than the tolerance (ϵ) and then the final ground state energy 〈*H*〉_min_ is obtained.

Here, we demonstrate a single qudit–based quantum processing unit (SQD-QPU) using OAM states of a single photon to estimate the ground state energy of the molecular system having a many-body interacting fermionic Hamiltonian as shown in Fig. 1. An OAM qudit ansatz state ∣ψ〉 is prepared from the fundamental Gaussian mode with (*l*, *p*) = (0,0), where *l* and *p* are integers corresponding to the azimuthal and radial indices, respectively, using the preparation SLM (SLM1) with a holographic image. Then, it is measured as intensity-modulated Gaussian mode corresponding to a set of Pauli measurements *P*_m_ = σ_i_ ⊗ σ_j_ ⊗ σ_k_⋯ using the measurement SLM (SLM2), as shown in Fig 1. Note that this multiple qubit Pauli measurement can be replaced by a simple single qudit measurement (*27*), and the SQD-QPU can efficiently construct a *d*-dimensional VQE using only a pair of SLMs (*28*–*30*). The expectation value of the Hamiltonian is estimated by a linear calculation of Pauli measurements, and a holographic image preparing a new ansatz qudit state with new parameters is generated in a single step by the classical optimizer of the classical processing unit (CPU).

**Fig. 2.**
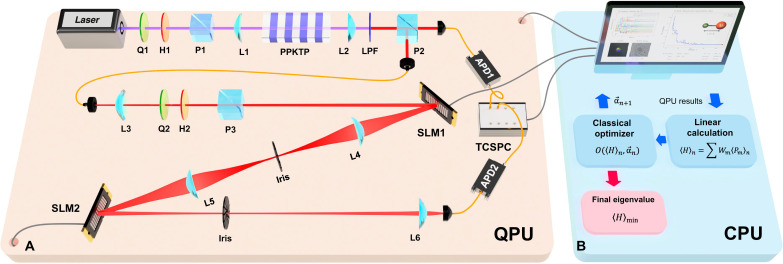
Experimental setup. (**A**) SQD-QPU with an OAM of a heralded single-photon. (**B**) CPU. A pair of photons is generated via type-II spontaneous parametric down-conversion process, and the signal photon goes directly into the APD1 for heralding, and the idler photon goes into the APD2 after undergoing the SQD-QPU. In SQD-QPU, the initial state of a fundamental Gaussian mode is converted to an OAM ansatz qudit state $|\psi \left(\alpha_{n}\right)\rangle$ using the SLM1, and a set of Pauli measurements is performed using the SLM2. The CPU estimates the lowest eigenvalue of the Hamiltonian *H* corresponding to the ground state energy and updates a set of new parameters $\alpha_{n+1}$ by the classical optimizer. (H, half-wave plate; Q, quarter-wave plate; P, polarizing beam splitter; L, lens; LPF, long pass filter; SLM, spatial light modulator; APD, avalanche photodiode; TCSPC, time-correlated single photon counter; PPKTP, periodically poled potassium titanyl phosphate.)

Our experimental setup for SQD-VQE is illustrated in Fig. 2. A heralded single-photon state is prepared by pumping a 20-mm-long periodically poled potassium titanyl phosphate crystal with a continuous wave diode laser via type-II spontaneous parametric down conversion (SPDC) process (*18*). Here, an OAM qudit can be efficiently implemented by encoding it in the LG modes using the SLM1. See Materials and Methods for detailed information on how we generate SLM images. A horizontally polarized single photon with a fundamental Gaussian mode propagates to the SLM1 and an ansatz qudit state $|\psi \left(\alpha_{n}\right)\rangle$ is generated by diffraction. The single-photon state is projected into a Pauli string basis using the measurement SLM (SLM2) and a single-mode optical fiber by far-field two-dimensional Fourier transform (*31*, *32*), as shown in Fig. 2A. The expectation value of the Hamiltonian is computed by performing linear calculations through the CPU. The classical optimizer updates the next parameters $\alpha_{n+1}$ that minimize the expectation value of the Hamiltonian in the CPU as shown in Fig. 2B. Here, we use the gradient-free COBYLA optimizer, which provides lower iteration and a higher success probability among the various classical optimizers (*5*). Last, when the tolerance value of $|\alpha_{n+1}-\alpha_{n}|$ becomes less than 0.01, the SQD-VQE program systematically stops and automatically returns the minimized eigenvalue of the Hamiltonian 〈*H*〉_min_. See Supplementary Note for the detailed information on our experimental setup and procedure.

**Fig. 3.**
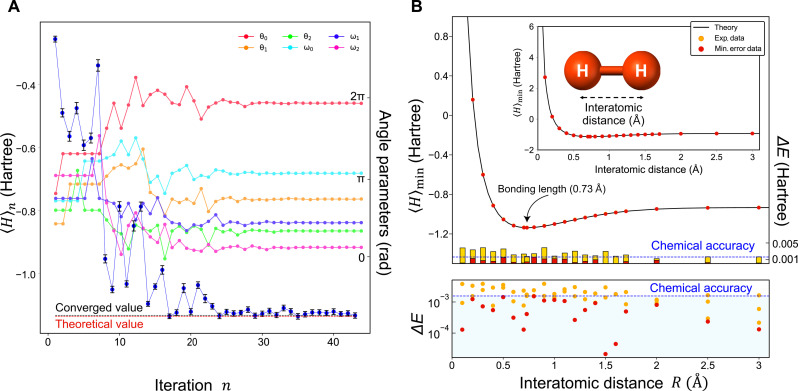
Experimental results for H_2_. (**A**) Iterative optimization results of the ground state energy 〈*H*〉 of H_2_ at interatomic distance *R* = 0.73 Å, which is the bonding length of H_2_. The blue circles, the red dashed line, and the black dashed line correspond to the experimentally estimated ground state energy at each iteration *n*, the theoretical ground state energy, and the converged ground state energy, respectively. The estimated ground state energy converges to a certain value with 0.0004 Hartree energy difference close to the theoretical value as the number of iterations *n* increases. The black error bars of the measured energy represent one standard deviation obtained by performing a Monte Carlo simulation 1000 times. (Background: Experimental results on six angle parameters that control the ansatz qudit state at each iteration. The amount of change on the angle parameters gradually decreases as the number of iterations *n* increases.) (**B**) Estimated ground state energy of H_2_ with respect to the interatomic distance *R*. Δ*E* corresponds to the difference between the calculated and the experimentally obtained ground state energies. The blue dashed line corresponds to chemical accuracy. We perform experiment at each interatomic distance multiple times, and the red circles correspond to the best result with the minimum energy difference Δ*E*, while the orange circles correspond to the other data. Note that the best data at each interatomic distance can achieve chemical accuracy, which is approximately 0.0016 Hartree energy difference.

We first consider a problem to find the ground state energy of H_2_ corresponding to a four-dimensional system to verify the performance of our SQD-VQE. The Hamiltonians that we used are generated by Qiskit Nature library (*8*, *33*). Note that the H_2_ ground state energy problem corresponds to a two-qubit problem. It is known that H_2_ has the minimum energy at the interatomic distance *R* = 0.73 Å, which is the bonding length of H_2_. The initial angle parameters $\alpha_{0}=\left(\theta_{0},\omega_{0}\right)$ start with arbitrary values. Figure 3A shows that the six angle parameters of four-dimensional ansatz qudit state at *R* = 0.73 Å is updated at each iteration. See Materials and Methods for the detailed information about these six angle parameters representing an ansatz state. The experimentally obtained ground state energy converges to the theory value (red dashed line) as the number of iterations increases. The result of SQD-VQE faithfully follows the objective of VQE to minimize the Rayleigh exponent based on the variational principle to give the minimized expectation energy at the desired interatomic distance.

Figure 3B shows the estimated ground state energy of H_2_ as a function of the interatomic distance *R*. Figure 3B inset shows the results including the total estimated ground state energy values for the experiment of H_2_ for the total interatomic distance. In Fig. 3B, both of the red and yellow bars show the difference between the experimental results and the theoretical value, and they correspond to the maximum and minimum energy differences, respectively. The bottom of Fig. 3B shows the log scale energy difference, and the blue dashed line is for chemical accuracy. It is clearly shown that the ground state energy of H_2_ estimated by SQD-VQE converges to the theoretical value. We emphasize that the experimentally estimated values deviate from the theoretical values by ∼0.00146 ± 0.000993 Hartree on average, and this is smaller than chemical accuracy, which is used to describe an accuracy in theoretical calculations of molecular properties or reactions, 4 kJ/mol (approximately 0.0016 Hartree), even without using any error-mitigation process. The average number of iterations to converge is ∼48 ± 6.8.

**Fig. 4.**
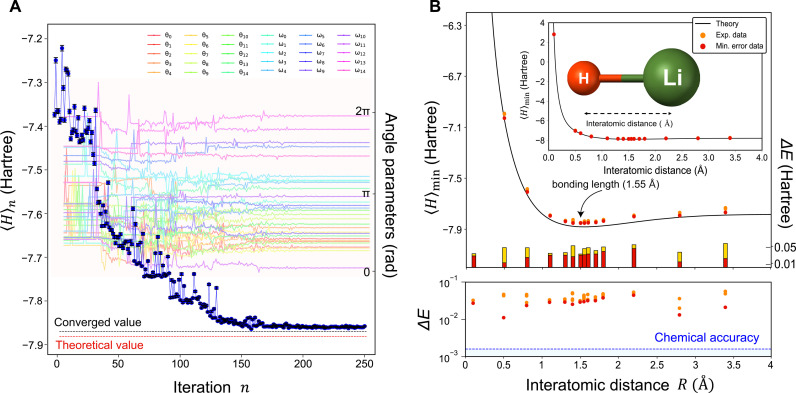
Experimental results for LiH. (**A**) Iterative optimization results of the ground state energy 〈*H*〉 of LiH at interatomic distance *R* = 1.55 Å. The blue circles, the red dashed line, and the black dashed line correspond to the experimentally estimated ground state energy at each iteration *n*, the theoretical ground state energy, and the converged ground state energy, respectively. The estimated ground state energy converges to a certain value with 0.029 Hartree energy difference close to the theoretical value as the number of iterations *n* increases. The black error bars of the measured energy represent one SD obtained by performing a Monte Carlo simulation 1000 times. (Background: Experimental results on 30 angle parameters that control the ansatz qudit state at each iteration. The amount of change on the angle parameters gradually decreases as the number of iterations *n* increases.) (**B**) Estimated ground state energy of LiH with respect to the interatomic distance *R*. Δ*E* corresponds to the difference between the calculated and the experimentally obtained ground state energies. The blue dashed line corresponds to chemical accuracy. We perform experiment at each interatomic distance multiple times and the red circles correspond to the best result with the minimum energy difference Δ*E* while the orange circles correspond to the other data.

We further evaluate the ground state energy of LiH with a 16-dimensional OAM qudit. The bonding length of LiH is *R* = 1.55 Å. Figure 4A shows how 30 angle parameters describing a 16-dimensional ansatz qudit state at *R* = 1.55 Å evolve at each iteration. The average number of iterations to converge to one minimum expectation value in our experiment is ∼242 ± 29.3, which is about five times larger than the case of H_2_. Figure 4B shows our experimental results of SQD-VQE for LiH. For LiH, our experimental results do not achieve the chemical accuracy, and an average difference with theoretical values is ∼0.036 ± 0.0098 Hartree. We attribute the experimental errors to the non-unity purity and state fidelity of our OAM states mainly caused by imperfect optical alignment, and we believe that they are not fundamental limit on our proposed scheme but can be further improved. We performed our SQD-VQE for other molecular systems such as He–H^+^ and LiH provided by OpenFermion library from Google Quantum AI and provided our experimental results on these problems in the Supplementary Materials.

## DISCUSSION

In summary, we have demonstrated SQD-VQE using a single qudit encoded in the OAM of a single photon. High-dimensional LG modes were efficiently generated and measured by displaying holographic images in SLMs. Our proposed SQD-VQE system consists of a pair of SLMs for state preparation and measurement of an OAM qudit, which provides more resource-efficient quantum processor configuration than existing VQE systems (*34*). We also estimated the ground state energy of the H_2_ and LiH corresponding to 4- and 16-dimensional qudit systems, respectively, in the SQD-VQE and confirmed that it successfully converged to the theoretical value. We emphasize again that we achieved the chemical accuracy for estimating the ground state of H_2_ even without using any error-mitigation process. Hence, our proposed SQD-VQE is expected to provide a promising approach for performing advanced quantum computation and information processing tasks.

We believe that our results on SQD-VQE might replace the existing VQE exploiting multiple qubits and further open a pathway of toward a photonic qudit-based quantum computer. OAM states are characterized by their spatial modes, and local perturbations in one OAM mode may not affect other OAM modes, making more stable quantum computation and simulation despite certain environmental disturbances (*35*, *36*). In addition, OAM qudit has the Hilbert space spanned by an infinite OAM basis, which results in higher quantum information density and increased computational power to make it suitable for solving more complex eigenvalue problems (*37*–*41*). In addition, by leveraging multi-qudit quantum gates (*16*, *42*, *43*), our method can be further extended to multi-qudit–based VQE, which can estimate the ground energy of more complex molecules in quantum chemistry (*44*–*46*). We further note that the dimension of a qudit can also be extended by combining various degrees of freedom of a single photon such as time bin, polarization, path, and frequency (*47*, *48*). There are numerous applications of our SQD-VQE including designing and analyzing new drugs, materials, and catalysts (*49*). Our approach can also be widely used to the fields of quantum chemistry and computation, such as quantum machine learning and optimization (*50*).

## MATERIALS AND METHODS

### Generation of SLM images

Here, we use a method to efficiently prepare and measure OAM states by displaying holographic images on SLMs. Creating a specific OAM state of a single photon requires holographic images including diffraction grating, phase encoding, and amplitude encoding techniques. Note that the LG modes have both azimuthal and radial indices *l* and *p*, respectively (*51*); however, we only use the *l* index. In our experiment, the qudit basis ∣*i*〉 is encoded as the following

$$l=\left\{ \begin{array}{cc} \pm 1,\pm 2,\cdots ,\pm d/2 & \text{if}\;d\;\text{is}\;\text{even},\\ 0,\pm 1,\pm 2,\cdots ,\pm \left(d-1\right)/2 & \text{if}\;d\;\text{is}\;\text{odd} \end{array}\right.$$ (4)

for example, ∣0〉 ≡ ∣−8〉*_l_*, ∣1〉 ≡ ∣−7〉*_l_*,⋯,∣15〉 ≡ ∣8〉*_l_* in 16 dimensions. The LG mode of the desired *l* value can be generated using phase encoding. However, when the first generated mode is reflected from the SLM, it overlaps with the fundamental Gaussian mode, which has *l* = 0 value, thus grating for diffraction is required (*52*). To equalize the intensity of the beam diffracted from grating, amplitude encoding is needed and then SLM image can be generated finally (*21*). See the Supplementary Materials for detailed information on the generation method we used.

When the fundamental Gaussian mode is diffracted from the holographic image, a desired qudit basis state ∣*i*〉 with a specific OAM value *l* can be efficiently prepared. In the same way, an arbitrary superposition of OAM basis state ∣ψ〉 = ∑*_i_c_i_*∣*i*〉 with ∑*_i_*∣*c_i_*∣^2^ = 1 can be efficiently realized by displaying holographic images combining different OAM values.

### Ansatz state representation

To update parameters by COBYLA, the ansatz state should be parameterized as real numbers. Arbitrary *d*-dimensional ansatz qudit can be expressed by 2*d*-2 angle parameters (*53*). The four-dimensional ansatz state $|\psi \left(\alpha_{n}\right)\rangle$ can be represented by six angle parameters that consist of elevation and azimuthal angles (θ_*n*,0∼2_ ∈ [0, π), ω_*n*,0∼2_ ∈ [0,2π)) as the following

$$|\psi (\alpha_{n})\rangle =\left(\begin{array}{c} \alpha_{n,0}\\ \alpha_{n,1}\\ \alpha_{n,2}\\ \alpha_{n,3} \end{array}\right)=\left(\begin{array}{c} \text{cos}\;\left(\frac{\theta_{n,0}}{2}\right)\;\text{cos}\;\left(\frac{\theta_{n,1}}{2}\right)\\ \text{cos}\;\left(\frac{\theta_{n,0}}{2}\right)\;\text{sin}\;\left(\frac{\theta_{n,1}}{2}\right)e^{i\omega_{n,1}}\\ \text{sin}\;\left(\frac{\theta_{n,0}}{2}\right)\;\text{cos}\;\left(\frac{\theta_{n,2}}{2}\right)e^{i\omega_{n,0}}\\ \text{sin}\;\left(\frac{\theta_{n,0}}{2}\right)\;\text{sin}\;\left(\frac{\theta_{n,2}}{2}\right)e^{i\left(\omega_{n,0}+\omega_{n,2}\right)} \end{array}\right)$$ (5)

See the Supplementary Materials for 16-dimensional ansatz state representation we used. The ansatz qudit state, starting from an arbitrary state, updates the new parameters $\left(\alpha_{n+1}\right)$ via the classical optimizer at every iteration. Note that this process completes the ansatz qudit state preparation and does not include additional quantum gate operations. After the iterative process, it converges to the desired eigenvector that minimizes the eigenvalues by the variational principle.

## Supplementary Materials

### The PDF file includes:

Sections S1 and S2 Tables S1 to S6 Figs. S1 to S11 Legends for movies S1 to S4 References
