# Supplementary material for: Qudit-based variational quantum eigensolver using photonic orbital angular momentum states
Source: Sci Adv. 2024 Oct 23;10(43):eado3472. doi: 10.1126/sciadv.ado3472 (PMC11498208; doi:10.1126/sciadv.ado3472)
Supplement: Supplementary file 1 — Sections S1 and S2 Tables S1 to S6 Figs. S1 to S11 Legends for movies S1 to S4 References [file sciadv.ado3472_sm.pdf]

Science Advances
AAAS

Supplementary Materials for

Qudit-based variational quantum eigensolver using photonic orbital angular momentum states

Byungjoo Kim *et al.*

Corresponding author: Hyang-Tag Lim, hyangtag.lim@kist.re.kr

Sci. Adv. **10**, eado3472 (2024)
DOI: 10.1126/sciadv.ado3472

The PDF file includes:

Sections S1 and S2
Tables S1 to S6
Figs. S1 to S11
Legends for movies S1 to S4
References

Other Supplementary Material for this manuscript includes the following:

Movies S1 to S4

1 Orbital Angular Momentum

1.1 Experimental setup for SQD-VQE

A detailed experimental setup for our single-qudit-based variational quantum eigensolver (SQD-VQE) using orbital angular momentum (OAM) of a single-photon is shown in Figure S1. A 20-mm-long periodically-poled potassium titanyl phosphate (PPKTP) is pumped by a 401 nm continuous wave (CW) single-mode diode laser with free-space power of 47 mW. Then, a pair of photons are probabilistically generated via type-II spontaneous parametric down conversion (SPDC) process. The down-converted signal photon directly goes to the APD1 and the other idler photon goes to the OAM setup part. The polarization state of an idler photon is set to the horizontal polarization state using a set of wave plates and a polarizing beam splitter (PBS) and then idler photon is sent to an OAM single qudit-based quantum process unit (SQD-QPU). Note that the light incoming through a set of waveplates should be aligned with the center of the first state preparation spatial light modulator (SLM, Holoeye Pluto-2.1) used to prepare an arbitrary ansatz qudit state. Holographic images are displayed in SLMs for LG modes' image implementation. By adding a blazed diffraction grating pattern to a holographic image, desired high-quality LG modes with high diffraction efficiency can be generated and measured at the first-order diffraction beam. After the SLM1, the prepared ansatz qudit state propagates through the lens-iris-lens system, the so-called $4f$ system *(31)*. The reflected and diffracted beams are filtered through the iris located at the center of the SQD-QPU for blocking all the other diffraction beams except the first-order diffraction b eam. The generated high-dimensional quantum state is imaged through aligned $4f$ system in a diagonal optical path to the measurement SLM (SLM2) where a set of Pauli measurements images are displayed. From the SLM2 images, the far-field distribution becomes a polynomial Gaussian function given by a two-dimensional Fourier transform. Then it is coupled to a single-mode fiber with filtering to the underlying

Gaussian mode as a signal via an objective lens. For measuring two-photon coincidence events between APD1 and APD2, we use a time-correlated single photon counter (TCSPC) with 2 ns coincidence time window.

1.2 Crosstalk matrix of Laguerre-Gaussian modes

The crosstalk matrix is a $d \times d$ matrix that verifies the preparation and measurement characteristics of Laguerre-Gaussian (LG) modes *(19)*. Each element of the crosstalk matrix is a measured value between two OAM modes $|\langle\psi_m|\psi_p\rangle|^2$, where $|\psi_p\rangle$ is the prepared eigenstates and $\langle\psi_m|$ is the measured eigenstates. The elements of the crosstalk matrix can theoretically be expected to be zero between orthogonal modes and a maximum value between the same modes. To minimize crosstalk between adjacent orthogonal modes in the experiment, we need precise optical alignment. If there is slight misalignment in the optical components, the off-diagonal elements of the crosstalk matrix will appear immediately, requiring more fine optical alignment in higher dimensions. We organize one column of the crosstalk matrix by preparing the eigenstates on the SLM1 and measuring all the eigenstates on the SLM2. Then, the next eigenstate is displayed on the SLM1 and all eigenstate measurements are repeated on the SLM2. In the 4f system, the same LG modes are converted into Gaussian beams by two-dimensional Fourier transform. The visibility V is defined as a measure of how well spatial LG modes are generated and measured and it is given by

$$V = \frac{\sum_{i=1}^{d} N_{ii}}{\sum_{i,j=1}^{d} N_{ij}}, \tag{1}$$

where $\sum_{i=1}^{d} N_{ii}$ is the sum of the diagonal elements of the crosstalk matrix, and $\sum_{i,j=1}^{d} N_{ij}$ is the sum of all coincidences of the elements of the crosstalk matrix for prepared LG modes i and measured LG modes j *(19)*. We obtained visibility $V \geq 0.973$ as shown in Figure S2 from the measured crosstalk matrix in 4-dimension and $V \geq 0.915$ in 16-dimension as shown in

Figure S3. We confirmed the small crosstalk between adjacent orthogonal LG modes (Almost negligible counts between two distant LG modes). Nevertheless this nonzero crosstalk can cause experimental errors on generating and measuring LG modes, and we expect that resolving this issue will reduce errors on the ground state energy of the molecule.

1.3 Generation of SLM images

The manipulation of OAM qudit states can be achieved using SLMs, cylindrical lenses, spiral phase plates, holographic gratings, metamaterials, and Q-plates *(20)*. Among them, a method utilizing SLMs simplifies the design and implementation of state preparation and measurement, thereby reducing the complexity and resource requirements of quantum computing systems. It requires three steps for generating images to implement OAM modes. First of all, the phase of LG modes is encoded as shown in Figure S4**A**. LG mode is the set of solutions of paraxial Helmholtz equation in cylindrical coordinates as the following *(54)*:

$$LG_p^l(\rho,\varphi,z) = \frac{w_0}{w(z)}\sqrt{\frac{2p!}{\pi(|l|+p)!}}\left(\frac{\sqrt{2}\rho}{w(z)}\right)^{|l|} L_p^{|l|}[2(\frac{\rho}{w(z)})^2]e^{i(2p+|l|+1)\xi(z)}e^{-\left(\frac{\rho}{w(z)}\right)^2}e^{\frac{-ik\rho^2}{2R(z)}}e^{-il\varphi}. \tag{2}$$

where w_0 is beam waist, $w(z)$ is $\sqrt{1+(\frac{z_R}{z})^2}$, $\xi(z)$ is arctan($\frac{z}{z_R}$), $R(z)$ is $z[1+(\frac{z}{z_R})^2]$ z_R is $\frac{\pi w_0^2}{\lambda}$, λ is wavelength. A density matrix for an arbitrary state can be converted to an image by projection of the LG mode basis and its phase encoding. However, in this case, only the phase encoded beam is overlapped with the reflected fundamental Gaussian mode with $l = 0$. Thus, blazed grating should be added as shown in Figure S4**B**. The grating like a saw can strengthen the first order diffraction *(52)*. The grating which is decided by step size (L) and the number of steps (N) affects the diffraction strength and angle. The L and N are both selected as eight in our experimental setup. Finally, the intensity masking is required for the equal diffracted beam intensity as shown in Figure S4**C**. The hologram pattern should be selected to give an equivalent transverse electric field in the first order diffraction. The pattern can be decided by an exact

solution for the boundary condition, such as quality and resolution of SLM's pixel which can control the phase delay *(21, 31)*. Then the final SLM image for LG mode can be obtained as shown in Figure S4**D**. Note that for generating an SLM image for measurement, one should consider two additional factors that the phase should become complex conjugate compared to the state preparation case, and the direction of the blazed grating should be opposite to the state preparation case.

2 Variational quantum eigensolver

2.1 Hamiltonian construction and representation

A key problem in quantum chemistry is to calculate the lowest energy eigenvalues of the Hamiltonian of a molecular electronic structure using the Rayleigh-Ritz variational principle *(25)* and it is known that most properties of interest in a molecule or material are determined from the eigenvalues of the Hamiltonian.

The standard (*ab initio*) molecular Hamiltonian used in quantum computation describes the nuclear charge Z_i for a set of electrons in system N, and the corresponding Hamiltonian is written as

$$H = \sum_i \frac{\nabla^2_{R_i}}{2M_i} - \sum_i \frac{\nabla^2_{r_i}}{2} - \sum_{i,j} \frac{Z_i}{\mid R_i - r_j \mid} + \sum_{i,j>i} \frac{Z_i Z_j}{\mid R_i - R_j \mid} + \sum_{i,j>i} \frac{1}{\mid r_i - r_j \mid} \tag{3}$$

where R_i, M_i, Z_i, and r_i correspond to the position, mass, and charge of the nuclei, and the position of the electrons, respectively.

The second quantization of the Hamiltonian is obtained by using the Born-Oppenhemier approximation, which assumes the nucleus with a fixed point charge. A basis ϕ_i to represent the wave function should be selected and the fermion creation operator $a_i^\dagger$ and annihilation operator

a_j of electrons are applied. Then the Hamiltonian can be written as

$$H = \sum_{pq} h_{pq} a_p^\dagger a_q + \frac{1}{2} \sum_{pqrs} h_{pqrs} a_p^\dagger a_q^\dagger a_r a_s, \tag{4}$$

with

$$h_{pq} = \int d\sigma d\phi_p^*(\sigma)(\frac{\nabla_r^2}{2} - \sum_i \frac{Z_i}{\mid R_i - r \mid})\phi_q(\sigma), \tag{5}$$

and

$$h_{pqrs} = \int d\sigma_1 d\sigma_2 \frac{\phi_p^*(\sigma_1)\phi_q^*(\sigma_2)\phi_r(\sigma_1)\phi_s(\sigma_2)}{\mid r_1 - r_2 \mid}, \tag{6}$$

where the indices p, q, r, and s stand for the four possible states in Hilbert space. Note that σ_i is now a spatial and spin coordinate with $\sigma_i = (r_i, s_i)$, and the standard anti-commutation relations that determine the action of $a_i^\dagger$ and a_j are $\{a_i^\dagger, a_j\} = \delta_{ij}$ and $\{a_i^\dagger, a_j^\dagger\} = \{a_i, a_j\} = 0$.

Finally, the second quantized Hamiltonian needs to be mapped to qubits for implementation in a quantum system. The most representative mapping method is the Jordan-Wigner transform, which results in the form of a Pauli string. The fermion creation operator $a_i^\dagger$ and annihilation operator a_j are mapped as:

$$\hat{a}_j^\dagger \longrightarrow \frac{X_j - iY_j}{2} \otimes Z_0 \otimes \cdots \otimes Z_{j-1}, \tag{7}$$

and

$$\hat{a}_j \longrightarrow \frac{X_j + iY_j}{2} \otimes Z_0 \otimes \cdots \otimes Z_{j-1}, \tag{8}$$

where X_j, Y_j, and Z_j are Pauli operators acting on the jth qubit.

Therefore, the expectation value of the second quantized Hamiltonian can be efficiently measured and computed in a system since the mapped Hamiltonian satisfies the requirement that it is the sum of tensor products of Pauli operators.

In this work, the molecular Hamiltonian is calculated using STO-3G basis *(8)*, and the Python Openfermion library is used to generate the one- and two-electron integrals *(55)*. The STO-3G minimum basis, which is widely used in quantum chemistry due to its effectiveness in producing electronic integrals, is created by fitting three Gaussians to Slater atomic orbitals.

2.2 Ansatz qudit state preparation

Arbitrary d-dimensional ansatz qudit can be expressed in $2d$-2 angle parameters, which consists of elevation angle (θ) and azimuthal angle (ω) *(53)*. Then an arbitrary 16-dimensional ansatz qudit state can be represented as the following:

$$|\psi(\boldsymbol{\alpha}_n)\rangle = \begin{pmatrix} \alpha_{n,0} \\ \alpha_{n,1} \\ \alpha_{n,2} \\ \alpha_{n,3} \\ \alpha_{n,4} \\ \alpha_{n,5} \\ \alpha_{n,6} \\ \alpha_{n,7} \\ \alpha_{n,8} \\ \alpha_{n,9} \\ \alpha_{n,10} \\ \alpha_{n,11} \\ \alpha_{n,12} \\ \alpha_{n,13} \\ \alpha_{n,14} \\ \alpha_{n,15} \end{pmatrix} = \begin{pmatrix} \cos(\frac{\theta_{n,0}}{2})\cos(\frac{\theta_{n,1}}{2})\cos(\frac{\theta_{n,2}}{2})\cos(\frac{\theta_{n,3}}{2}) \\ \cos(\frac{\theta_{n,0}}{2})\cos(\frac{\theta_{n,1}}{2})\cos(\frac{\theta_{n,2}}{2})\sin(\frac{\theta_{n,3}}{2})e^{i\omega_{n,3}} \\ \cos(\frac{\theta_{n,0}}{2})\cos(\frac{\theta_{n,1}}{2})\sin(\frac{\theta_{n,2}}{2})\cos(\frac{\theta_{n,4}}{2})e^{i\omega_{n,2}} \\ \cos(\frac{\theta_{n,0}}{2})\cos(\frac{\theta_{n,1}}{2})\sin(\frac{\theta_{n,2}}{2})\sin(\frac{\theta_{n,4}}{2})e^{i(\omega_{n,2}+\omega_{n,4})} \\ \cos(\frac{\theta_{n,0}}{2})\sin(\frac{\theta_{n,1}}{2})\cos(\frac{\theta_{n,5}}{2})\cos(\frac{\theta_{n,6}}{2})e^{i\omega_{n,1}} \\ \cos(\frac{\theta_{n,0}}{2})\sin(\frac{\theta_{n,1}}{2})\cos(\frac{\theta_{n,5}}{2})\sin(\frac{\theta_{n,6}}{2})e^{i(\omega_{n,1}+\omega_{n,6})} \\ \cos(\frac{\theta_{n,0}}{2})\sin(\frac{\theta_{n,1}}{2})\sin(\frac{\theta_{n,5}}{2})\cos(\frac{\theta_{n,7}}{2})e^{i(\omega_{n,1}+\omega_5)} \\ \cos(\frac{\theta_{n,0}}{2})\sin(\frac{\theta_{n,1}}{2})\sin(\frac{\theta_{n,5}}{2})\sin(\frac{\theta_{n,7}}{2})e^{i(\omega_{n,1}+\omega_{n,5}+\omega_{n,7})} \\ \sin(\frac{\theta_{n,0}}{2})\cos(\frac{\theta_{n,8}}{2})\cos(\frac{\theta_{n,9}}{2})\cos(\frac{\theta_{n,10}}{2})e^{i\omega_{n,0}} \\ \sin(\frac{\theta_{n,0}}{2})\cos(\frac{\theta_{n,8}}{2})\cos(\frac{\theta_{n,9}}{2})\sin(\frac{\theta_{n,10}}{2})e^{i(\omega_{n,0}+\omega_{n,10})} \\ \sin(\frac{\theta_{n,0}}{2})\cos(\frac{\theta_{n,8}}{2})\sin(\frac{\theta_{n,9}}{2})\cos(\frac{\theta_{n,11}}{2})e^{i(\omega_{n,0}+\omega_{n,9})} \\ \sin(\frac{\theta_{n,0}}{2})\cos(\frac{\theta_{n,8}}{2})\sin(\frac{\theta_{n,9}}{2})\sin(\frac{\theta_{n,11}}{2})e^{i(\omega_{n,0}+\omega_{n,9}+\omega_{n,11})} \\ \sin(\frac{\theta_{n,0}}{2})\sin(\frac{\theta_{n,8}}{2})\cos(\frac{\theta_{n,12}}{2})\cos(\frac{\theta_{n,13}}{2})e^{i(\omega_{n,0}+\omega_{n,8})} \\ \sin(\frac{\theta_{n,0}}{2})\sin(\frac{\theta_{n,8}}{2})\cos(\frac{\theta_{n,12}}{2})\sin(\frac{\theta_{n,13}}{2})e^{i(\omega_{n,0}+\omega_8+\omega_{n,13})} \\ \sin(\frac{\theta_{n,0}}{2})\sin(\frac{\theta_{n,8}}{2})\sin(\frac{\theta_{n,12}}{2})\cos(\frac{\theta_{n,14}}{2})e^{i(\omega_{n,0}+\omega_8+\omega_{n,12})} \\ \sin(\frac{\theta_{n,0}}{2})\sin(\frac{\theta_{n,8}}{2})\sin(\frac{\theta_{n,12}}{2})\sin(\frac{\theta_{n,14}}{2})e^{i(\omega_{n,0}+\omega_{n,8}+\omega_{n,12}+\omega_{n,14})} \end{pmatrix}, \quad (9)$$

where $\theta \in [0, \pi)$, $\omega \in [0, 2\pi)$.

Note that due to the angular representation of the 16-dimensional ansatz qudit state, all normalized complex numbers can be effectively expressed with only 30 parameters, which is reduced from 32 parameters. The ansatz qudit state, starting from an arbitrary state, updates the new parameters ($\boldsymbol{\theta}, \boldsymbol{\omega}$) via the classical optimizer at every iteration. Note that this process completes the ansatz qudit state preparation and does not include additional quantum gate operations. The updated ansatz parameters are represented as a vector according to Eq. 9. This can be expressed as a superposition of various LG modes, where each element corresponds to the coefficient of an LG mode. Phase encoding with complex coefficients in LG mode, blazed

grating, and intensity masking techniques is combined to generate a single holographic image for the ansatz state, which is converted to a grayscale range of 0 to 255 integers and displayed on the SLM. The initial Gaussian mode is incident at the center of the generated holographic image, and the superposition of the desired LG mode field as an ansatz state is precisely obtained in the far field at the first diffracted beam. Through this process, we can encode the phase of the desired LG mode superposition state. After the iterative process, it converges to the desired eigenvector that minimizes the eigenvalues by the variational principle.

2.3 Pauli grouping and measurement.

By grouping the Pauli strings to Abelian group whose group operation is commutative, we can reduce the number of terms to be measured for computing the expectation value of the Hamiltonian. This reduction is attributed to the simultaneous diagonalization of the Abelian group of Pauli strings through a unitary rotation of the measurement basis *(3)*. The 100 Pauli matrices are generated for estimating the ground state energy of LiH using the Qiskit library *(33)*. They were grouped by Pennylane considering the commuting method (11 groups) and the Qubit-wise commuting method (27 groups) *(56)*. Both methods work on the same principle, but the Qubit-wise commuting method has more constraints than the commuting method *(3)*. Here, one group for LiH is composed of 16 Pauli measurement elements, and each group is calculated at once through a linear calculation after a set of Pauli measurements. In our experiment, the VQE results of two methods have almost same energy difference from the theoretical value. To reduce the measurement time and maintain the stability of the experimental setup, the commuting method, which provides fewer groups of Pauli measurements, was chosen as the grouping method. We provide the Pauli weight coefficients for four molecular models (H_2, LiH from Qiskit, He–H^+, LiH from openfermion) in Tables S1, S2, S3, and S4. The images for Pauli measurements can be generated by encoding the eigenvectors corresponding to the measure-

ment bases of the Pauli strings within the same group into LG modes and converting the phase information into grayscale. Figure S6 shows examples of Pauli measurement images for the He-H+ group, and each image was phase-encoded using the eigenvectors corresponding to the rows and columns in Table S5. For Group 1, these 4 images in the first row are displayed on the measurement SLM respectively, and we can obtain coincidence counts for each. The normalized coincidence counts C_1, C_2, C_3, and C_4 can be obtained by dividing each by the total coincidence count. The expectation values of Pauli strings in Group 1 can be linearly calculated using normalized coincidence counts; for example, $C_1+C_2+C_3-C_4$ for IZ. By multiplying the obtained expectation values of all the Pauli strings by their corresponding weight coefficients and performing a linear calculation, we can estimate the expectation value of the Hamiltonian on the CPU.

2.4 Classical optimizer

The classical optimizer's task is to minimize the eigenvalue by optimizing the parameters in the VQE. We conducted simulations with using six classical optimizers to select the most suitable one, including three gradient-free optimizers (COBYLA, Nelder-Mead, Powell) and three gradient-based optimizers (Conjugate Gradient, BFGS, SLSQP) provided by SciPy, an open-source library of python. The simulation was performed using the expectation value of the measurement result. The results of 100 simulations are shown in Figure S5. The vertical green line represents the theoretical value of the ground state energy of LiH, which is generated by Openfermionpsi4 *(55)* at the bonding length. The red bars represent the frequency of the converged energy. Among the classical optimizers, COBYLA (Constrained Optimization BY Linear Approximations) had the shortest convergence time of approximately 1.5 seconds, with the fewest iterations, and converged to the global minimum more frequently than most other cases. Since COBYLA also showed the best results in another study *(5)*, we decided to

use COBYLA, an optimization algorithm that iteratively solves problems by linearly approximating the objective function and constraints, dynamically adjusting the trust region to ensure convergence *(57)*, in our experiments.

2.5 Results of other eigenvalue problems for quantum chemistry

We estimated quantum chemistry problems for the Hamiltonian generated by Openfermion *(55)*, provided by Google Quantum AI, for two models: He–H^+ and LiH. He–H^+ can be represented as four-dimensional problem *(4, 5)*. The experimentally obtained ground state energy converges to the theory value (Red dotted line) as the number of iterations increases due to the update of the new angle parameters provided by the classical optimizer as shown in Figure S7**A**. Figure S7**A** shows that the six parameters of four-dimensional ansatz qudit state at $R = 0.9$ Å are updated at each iteration.

Figure S7**B** shows the estimated ground state energy of a He–H^+ as function of the total interatomic distance R. In Figure S7**B**, the yellow and red bars corresponds to the maximum and minimum energy differences, respectively. The red circles represent the best result which has the minimum difference with theorical value (ΔE) and orange circles are for all of the other data. The bottom of the Figure S7**B** shows the log scale energy difference and blue dotted line is for chemical accuracy. Figure S7**B** inset shows the results including the total estimated ground state energy values for the experiment of He–H^+ for the total interatomic distance. The average number of iterations to converge to one minimum expectation value in the experiment is $\sim 52 \pm 8.3$. It is clearly shown that the ground state energy of He–H^+ estimated by SQD-VQE converges to the theoretical value with a high success probability. Their mean of difference is $\sim\ 0.00151 \pm 0.000829$ Hartree and it is smaller than chemical accuracy which is used to describe accuracy without using any error-mitigation process.

We also estimate the ground state energy of LiH. The Hamiltonian of the openfermion-LiH

is different from Qiskit one, which is used in the main text. For the openfermion-LiH, the bonding length is $R = 1.51\ \mathring{A}$. Figure S8**A** also shows how 30 angle parameters describing a 16-dimensional ansatz qudit state at $R = 1.51\ \mathring{A}$ evolve at each iteration. The average number of iterations to converge to one minimum expectation value in our experiment is $\sim 210 \pm 23.5$. Figure S8**B** shows our experimental results of SQD-VQE for LiH. Note that our experimental results are very well matched to the theoretical curve. For LiH, two data points can achieve the chemical accuracy. Although we cannot achieve the chemical accuracy for the other interatomic distances, our results show that SQD-VQE can potentially achieve the chemical accuracy in 16 dimension case. The average difference with theoretical values is $\sim 0.0079 \pm 0.0064$ Hartree.

2.6 Measurement strategy

In our VQE experiment using OAM, when the SLM image is switched on a new measurement image, the previous image can disturb the new one as an afterimage. Thus the delay for SLM switching t_{delay} is needed. The delay should be minimized since it affects every iteration time t_n. Figure S9 is the outcome of the measurement used to establish the interval for image output to the SLM. We measured the time which takes for the SLM2 switching on from $\text{LG}_{l=8}$ to $\text{LG}_{l=1}$ until the measured counts are stabilized. The error bars correspond one standard deviation from 1000 measurements. We chose $t_{\text{delay}} = 0.2$ s for ensuring stable measurements. The integration time t_{int} of the measurement device was finally configured to 0.2 s. Hence, the time for iterations is approximately optimized to this for minimizing the total VQE running time.

$$t_n = N_{\text{g}} d(t_{\text{delay}} + t_{\text{int}}) + t_{\text{a}}, \tag{10}$$

where t_n is the time for iteration n and N_{g} is the number of Pauli groups, d is dimension. t_{a} is the time for generating ansatz image. The parameters that we use for four molecular models are shown as Table S6.

	Group 1 (2)		Group 2 (3)		
**R (Å)**	**II**	**XX**	**IZ**	**ZI**	**ZZ**
0.1	4.75665	0.15617	1.027315	-1.027315	-0.013867
0.2	2.011528	0.157973	0.930489	-0.930489	-0.013624
0.3	1.010182	0.160819	0.808649	-0.808649	-0.013288
0.4	0.460363	0.164515	0.688194	-0.688194	-0.012914
0.5	0.110647	0.16887	0.58308	-0.58308	-0.012516
0.6	-0.125165	0.173731	0.490138	-0.490138	-0.012064
0.7	-0.287945	0.179001	0.420456	-0.420456	-0.011507
0.73	-0.326386	0.180653	0.401061	-0.401061	-0.011314
0.8	-0.401741	0.184627	0.359959	-0.359959	-0.01081
0.9	-0.482309	0.190572	0.309787	-0.309787	-0.009969
1	-0.540066	0.196791	0.267529	-0.267529	-0.009015
1.1	-0.581742	0.203222	0.231396	-0.231396	-0.007995
1.2	-0.61169	0.209791	0.20019	-0.20019	-0.006962
1.3	-0.632857	0.216417	0.173108	-0.173108	-0.005962
1.4	-0.647375	0.223022	0.149568	-0.149568	-0.005031
1.5	-0.65686	0.229536	0.129101	-0.129101	-0.004189
1.6	-0.662563	0.235901	0.111309	-0.111309	-0.003448
1.7	-0.665457	0.242073	0.095845	-0.095845	-0.002808
2	-0.663968	0.259138	0.060628	-0.060628	-0.001431
2.5	-0.649052	0.28221	0.027135	-0.027135	-0.000377
3	-0.633651	0.299212	0.011235	-0.011235	-0.000074

Table S1. **H_2 Pauli operators.**

**Group 1 (16)**		**Group 2 (14)**		**Group 3 (11)**		**Group 4 (11)**	
IIII	-6.996701	IIZX	-0.002953	ZXZI	-0.010690	XZYY	-0.007864
IIIZ	-0.092811	IIIX	-0.002953	IXZI	-0.010690	IIXX	-0.000767
IZXZ	-0.012782	IIZI	0.364916	IIZZ	-0.211881	IIYY	0.000767
IZXI	-0.012782	XZZX	0.002228	ZXII	0.002953	YYII	0.000767
IIXZ	0.019380	IZZI	0.113930	IXII	0.002953	ZXXX	-0.008506
IIXI	0.019380	IZZX	0.011936	ZIZI	-0.113827	ZXYY	0.008506
IZII	0.092811	IZIX	-0.011936	ZXIZ	0.011936	YYZZ	-0.002756
ZZIZ	0.056269	XIZX	-0.002228	IXIZ	0.011936	XZZZ	0.009002
ZZII	-0.211881	XZIX	-0.002228	ZXZZ	-0.001706	YYXX	-0.030852
ZZXZ	-0.009002	XIIX	0.002228	IXZZ	-0.001706	YYYY	0.030852
ZZXI	-0.009002	XZZI	0.010956	ZIZZ	0.060436	XZXX	0.007864
ZIII	-0.364913	XIZI	-0.010956				
IZIZ	-0.122697	XZII	-0.019380				
ZIXZ	0.010956	XIII	0.019380				
ZIXI	0.010956						
ZIIZ	0.113930						
**Group 5 (9)**		**Group 6 (7)**		**Group 7 (9)**		**Group 8 (6)**	
XXII	-0.000767	XXZX	0.008506	IZXX	0.032360	IZYY	-0.032360
IXXI	0.002228	YYZX	-0.008506	ZXZX	-0.003145	ZXIX	0.003145
XXXI	-0.007864	XXYY	-0.030852	IXZX	-0.003145	XXIZ	-0.032360
IXIX	0.003145	ZZZX	0.001706	ZZXX	0.002756	ZIZX	-0.010690
IXXX	-0.008506	ZZYY	-0.002756	ZIYY	0.034349	YYXI	0.007864
XIXI	0.006589	XXXZ	-0.007864	IZZZ	-0.056269	XIZZ	-0.009002
XXIX	-0.008506	YYXZ	0.007864	ZXXZ	0.002228		
XXXX	0.030852			IXXZ	0.002228		
XIXX	-0.007864			ZZZZ	0.084595		
**Group 9 (7)**		**Group 10 (6)**		**Group 11 (4)**			
IXYY	0.008506	YYIZ	0.032360	XZIZ	-0.012782		
YYIX	0.008506	ZXXI	0.002228	XIIZ	0.012782		
ZZIX	-0.001706	XXZZ	0.002756	ZIIX	0.010690		
XIXZ	0.006589	XZXZ	-0.006589	ZIXX	-0.034349		
XIYY	0.007864	XXZI	0.034349				
YYZI	-0.034349	XZXI	-0.006589				
ZZZI	-0.060436						

Table S2. **LiH-qiskit Pauli operators at bond distance (1.55 Å).**

	Group 1 (4)				Group 2 (2)		Group 3 (3)		Group 4 (1)
**R (Å)**	**II**	**IZ**	**ZI**	**ZZ**	**IX**	**ZX**	**XI**	**XZ**	**XX**
0.05	6.4665	-0.472	-0.472	0.0523	-0.0289	0.0289	-0.0289	0.0289	0.0269
0.15	1.2994	-0.4533	-0.4533	0.0288	-0.0293	0.0293	-0.0293	0.0293	0.051
0.25	0.324	-0.4129	-0.4129	0.0192	-0.0252	0.0252	-0.0252	0.0252	0.0612
0.35	-0.1106	-0.36	-0.36	0.0178	-0.0254	0.0254	-0.0254	0.0254	0.0637
0.45	-0.358	-0.3112	-0.3112	0.0196	-0.0281	0.0281	-0.0281	0.0281	0.0636
0.55	-0.5112	-0.2716	-0.2716	0.0232	-0.0318	0.0318	-0.0318	0.0318	0.0622
0.65	-0.6098	-0.2417	-0.2417	0.0281	-0.0356	0.0356	-0.0356	0.0356	0.0597
0.75	-0.6745	-0.22	-0.22	0.0341	-0.0392	0.0392	-0.0392	0.0392	0.0563
0.85	-0.7175	-0.2049	-0.2049	0.0411	-0.0423	0.0423	-0.0423	0.0423	0.0521
0.9	-0.7333	-0.1993	-0.1993	0.0449	-0.0436	0.0436	-0.0436	0.0436	0.0498
0.95	-0.7462	-0.1949	-0.1949	0.0488	-0.0446	0.0446	-0.0446	0.0446	0.0472
1.05	-0.7652	-0.1888	-0.1888	0.0571	-0.046	0.046	-0.046	0.046	0.0419
1.15	-0.7775	-0.1858	-0.1858	0.0657	-0.0463	0.0463	-0.0463	0.0463	0.0363
1.25	-0.7851	-0.185	-0.185	0.0743	-0.0456	0.0456	-0.0456	0.0456	0.0307
1.35	-0.7894	-0.1858	-0.1858	0.0826	-0.0438	0.0438	-0.0438	0.0438	0.0253
1.45	-0.7916	-0.1876	-0.1876	0.0905	-0.0413	0.0413	-0.0413	0.0413	0.0203
1.55	-0.7923	-0.19	-0.19	0.0977	-0.038	0.038	-0.038	0.038	0.0159
1.65	-0.792	-0.1926	-0.1926	0.1041	-0.0344	0.0344	-0.0344	0.0344	0.0122
1.75	-0.7911	-0.1954	-0.1954	0.1098	-0.0306	0.0306	-0.0306	0.0306	0.0091
1.85	-0.7898	-0.1981	-0.1981	0.1147	-0.0267	0.0267	-0.0267	0.0267	0.0067
1.95	-0.7882	-0.2007	-0.2007	0.1189	-0.023	0.023	-0.023	0.023	0.0048
2.05	-0.7866	-0.2031	-0.2031	0.1225	-0.0196	0.0196	-0.0196	0.0196	0.0034
2.15	-0.7849	-0.2054	-0.2054	0.1256	-0.0166	0.0166	-0.0166	0.0166	0.0024
2.25	-0.7832	-0.2074	-0.2074	0.1282	-0.0139	0.0139	-0.0139	0.0139	0.0016
2.35	-0.7816	-0.2094	-0.2094	0.1305	-0.0115	0.0115	-0.0115	0.0115	0.0011
2.45	-0.78	-0.2111	-0.2111	0.1326	-0.0095	0.0095	-0.0095	0.0095	0.0007
2.55	-0.7785	-0.2127	-0.2127	0.1344	-0.0078	0.0078	-0.0078	0.0078	0.0005
2.65	-0.7771	-0.2142	-0.2142	0.136	-0.0064	0.0064	-0.0064	0.0064	0.0003
2.75	-0.7758	-0.2156	-0.2156	0.1375	-0.0052	0.0052	-0.0052	0.0052	0.0002
2.85	-0.7745	-0.2169	-0.2169	0.1388	-0.0042	0.0042	-0.0042	0.0042	0.0001
2.95	-0.7733	-0.2181	-0.2181	0.14	-0.0034	0.0034	-0.0034	0.0034	0.0001
3.05	-0.7722	-0.2192	-0.2192	0.1412	-0.0027	0.0027	-0.0027	0.0027	0.0001
3.15	-0.7712	-0.2203	-0.2203	0.1423	-0.0022	0.0022	-0.0022	0.0022	0.0
3.25	-0.7702	-0.2213	-0.2213	0.1432	-0.0017	0.0017	-0.0017	0.0017	0.0
3.35	-0.7693	-0.2222	-0.2222	0.1442	-0.0014	0.0014	-0.0014	0.0014	0.0
3.45	-0.7684	-0.223	-0.223	0.1451	-0.0011	0.0011	-0.0011	0.0011	0.0
3.55	-0.7676	-0.2239	-0.2239	0.1459	-0.0008	0.0008	-0.0008	0.0008	0.0
3.65	-0.7668	-0.2246	-0.2246	0.1467	-0.0007	0.0007	-0.0007	0.0007	0.0
3.75	-0.7661	-0.2254	-0.2254	0.1474	-0.0005	0.0005	-0.0005	0.0005	0.0
3.85	-0.7654	-0.2261	-0.2261	0.1481	-0.0004	0.0004	-0.0004	0.0004	0.0
3.95	-0.7647	-0.2267	-0.2267	0.1488	-0.0003	0.0003	-0.0003	0.0003	0.0

Table S3. **He–H^+ Pauli operators.**

Group 1 (11)		Group 2 (8)		Group 3 (8)	
IIII	-7.503816	IXZX	0.013347	IIIZ	0.159676
YYXX	-0.003056	ZXZX	-0.001574	ZXZX	0.011774
XYYX	0.003056	IXIX	0.011774	YZYZ	0.011774
YXXY	0.003056	IYZY	0.013347	XZXI	0.013347
XXYY	-0.03056	ZYZY	-0.001574	XIXI	-0.001574
IIZZ	0.123396	IYIY	0.011774	YZYI	0.013347
IZIZ	0.053521	IIZI	0.159676	YIYI	-0.001574
ZIIZ	0.056577	ZIII	-0.014048	IZII	-0.014048
IZZI	0.056577				
ZIZI	0.053521				
ZZII	0.084683				

Table S4. **LiH-openfermion Pauli operators at bond distance (1.51 Å).**

$\begin{pmatrix} 1 & 0 & 0 & 0 \\ 0 & 0 & 0 & 0 \\ 0 & 0 & 0 & 0 \\ 0 & 0 & 0 & 0 \end{pmatrix}$	$\begin{pmatrix} 0 & 0 & 0 & 0 \\ 0 & 1 & 0 & 0 \\ 0 & 0 & 0 & 0 \\ 0 & 0 & 0 & 0 \end{pmatrix}$	$\begin{pmatrix} 0 & 0 & 0 & 0 \\ 0 & 0 & 0 & 0 \\ 0 & 0 & 1 & 0 \\ 0 & 0 & 0 & 0 \end{pmatrix}$	$\begin{pmatrix} 0 & 0 & 0 & 0 \\ 0 & 0 & 0 & 0 \\ 0 & 0 & 0 & 0 \\ 0 & 0 & 0 & 1 \end{pmatrix}$
$\begin{pmatrix} 1/2 & 1/2 & 0 & 0 \\ 1/2 & 1/2 & 0 & 0 \\ 0 & 0 & 0 & 0 \\ 0 & 0 & 0 & 0 \end{pmatrix}$	$\begin{pmatrix} 1/2 & -1/2 & 0 & 0 \\ -1/2 & 1/2 & 0 & 0 \\ 0 & 0 & 0 & 0 \\ 0 & 0 & 0 & 0 \end{pmatrix}$	$\begin{pmatrix} 0 & 0 & 0 & 0 \\ 0 & 0 & 0 & 0 \\ 0 & 0 & 1/2 & 1/2 \\ 0 & 0 & 1/2 & 1/2 \end{pmatrix}$	$\begin{pmatrix} 0 & 0 & 0 & 0 \\ 0 & 0 & 0 & 0 \\ 0 & 0 & 1/2 & -1/2 \\ 0 & 0 & -1/2 & 1/2 \end{pmatrix}$
$\begin{pmatrix} 1/2 & 0 & 1/2 & 0 \\ 0 & 0 & 0 & 0 \\ 1/2 & 0 & 1/2 & 0 \\ 0 & 0 & 0 & 0 \end{pmatrix}$	$\begin{pmatrix} 0 & 0 & 0 & 0 \\ 0 & 1/2 & 0 & 1/2 \\ 0 & 0 & 0 & 0 \\ 0 & 1/2 & 0 & 1/2 \end{pmatrix}$	$\begin{pmatrix} 1/2 & 0 & -1/2 & 0 \\ 0 & 0 & 0 & 0 \\ -1/2 & 0 & 1/2 & 0 \\ 0 & 0 & 0 & 0 \end{pmatrix}$	$\begin{pmatrix} 0 & 0 & 0 & 0 \\ 0 & 1/2 & 0 & -1/2 \\ 0 & 0 & 0 & 0 \\ 0 & -1/2 & 0 & 1/2 \end{pmatrix}$
$\begin{pmatrix} 1/4 & 1/4 & 1/4 & 1/4 \\ 1/4 & 1/4 & 1/4 & 1/4 \\ 1/4 & 1/4 & 1/4 & 1/4 \\ 1/4 & 1/4 & 1/4 & 1/4 \end{pmatrix}$	$\begin{pmatrix} 1/4 & -1/4 & -1/4 & 1/4 \\ -1/4 & 1/4 & 1/4 & -1/4 \\ -1/4 & 1/4 & 1/4 & -1/4 \\ 1/4 & -1/4 & -1/4 & 1/4 \end{pmatrix}$	$\begin{pmatrix} 1/4 & -1/4 & 1/4 & -1/4 \\ -1/4 & 1/4 & -1/4 & 1/4 \\ 1/4 & -1/4 & 1/4 & -1/4 \\ -1/4 & 1/4 & -1/4 & 1/4 \end{pmatrix}$	$\begin{pmatrix} 1/4 & 1/4 & -1/4 & -1/4 \\ 1/4 & 1/4 & -1/4 & -1/4 \\ -1/4 & -1/4 & 1/4 & 1/4 \\ -1/4 & -1/4 & 1/4 & 1/4 \end{pmatrix}$

Table S5. **Outer product form of the eigenvectors of each Pauli string group for He-H+.**

Molcule	d	N_g	t_{delay}	t_{int}	t_{a}	Number of n
H_2	4	2	0.2	0.2	0.5	48 ± 6.8
He–H^+	4	4	0.2	0.2	0.5	52 ± 8.3
LiH-Qiskit	16	3	0.2	0.2	0.8	242 ± 29.3
LiH-Openfermion	16	11	0.2	0.2	0.8	210 ± 23.5

Table S6. **Time parameters for molecular models in our experiment.**

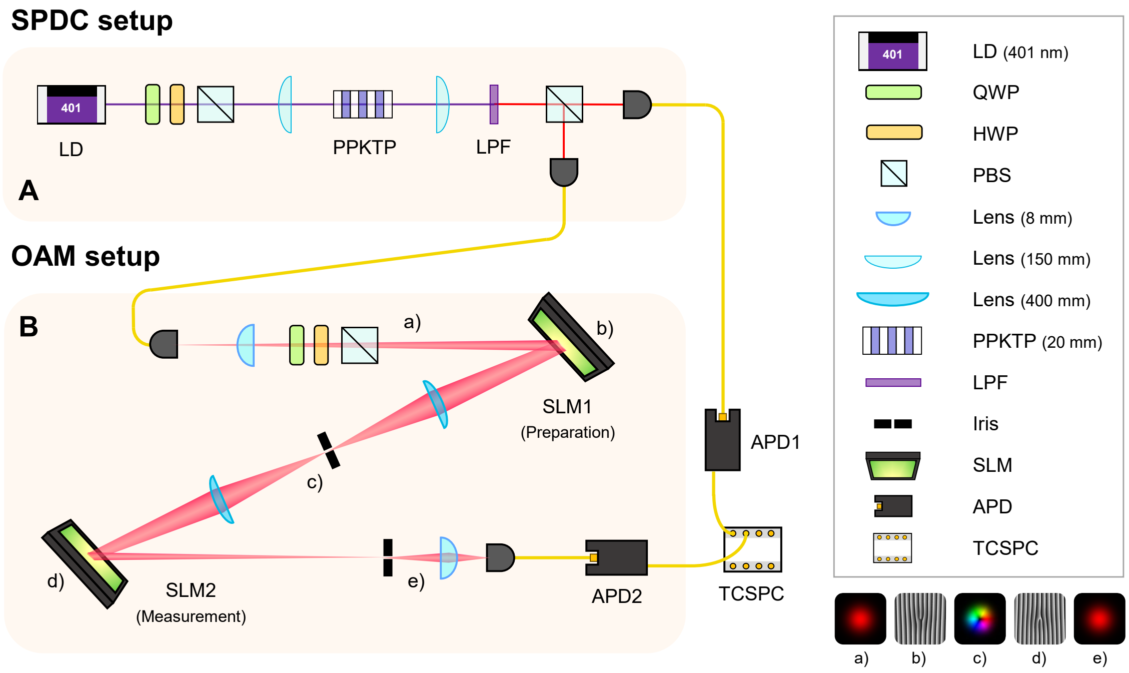

Figure S1. **Experimental setup for SQD-QPU. A,** SPDC setup for preparing a heralded single-photon state. **B,** OAM setup for state preparation and measurement. (LD, Laser Diode; HWP, Half-wave plate; QWP, Quarter-wave plate; PBS, Polarizing beam splitter; LPF, Long pass filter; SLM, Spatial light modulator; APD, Avalanche photodiode; TCSPC, Time-correlated single photon counter)

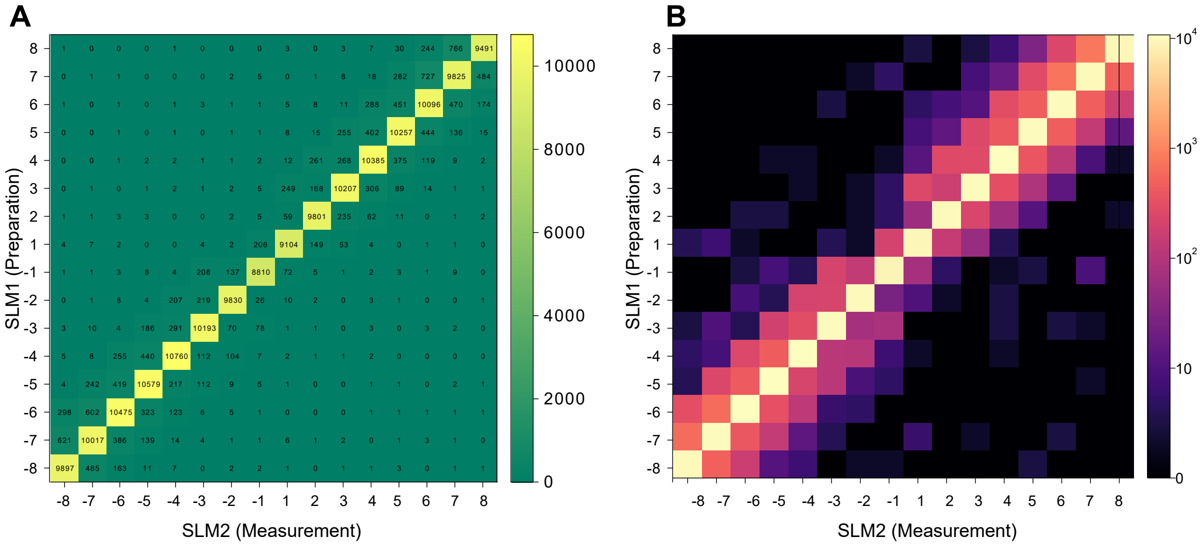

Figure S2. **Experimental** 4**-dimensional preparation-measurement crosstalk matrix. A,** linear and **B,** log scales.

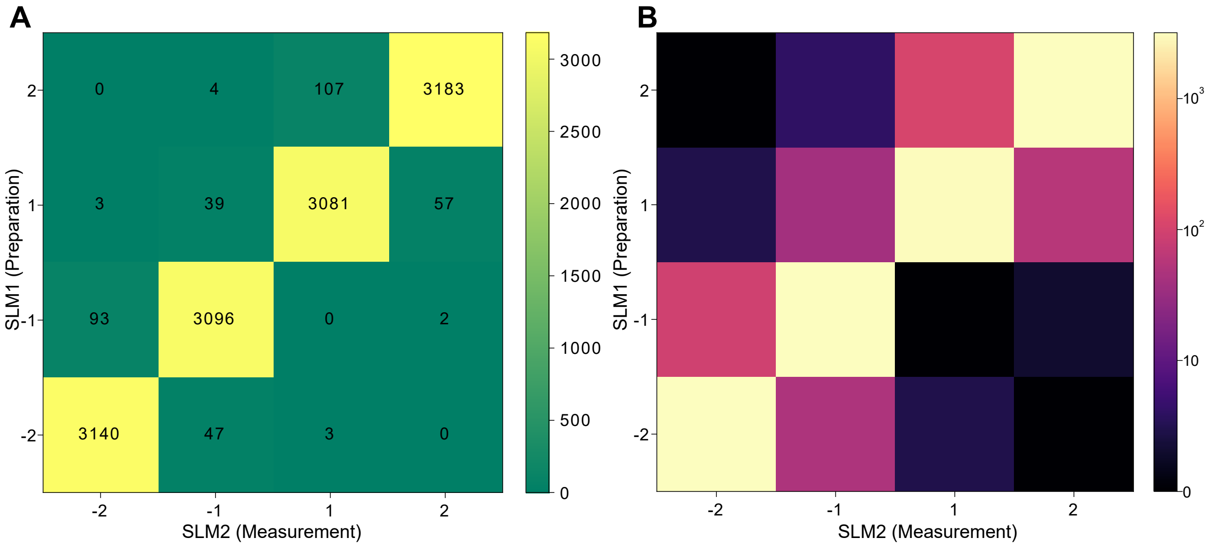

Figure S3. **Experimental** 16**-dimensional preparation-measurement crosstalk matrix. A,** linear and **B,** log scales.

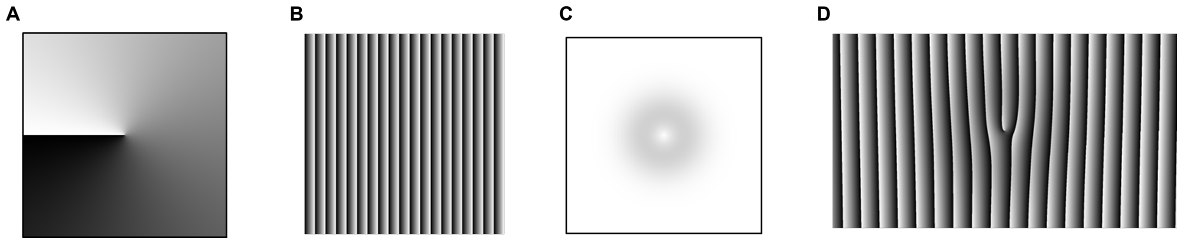

Figure S4. **Generation steps of an SLM image. A,** Laguerre Gaussian mode phase encoding. **B,** Blazed grating. **C,** Intensity masking. **D,** Final SLM image.

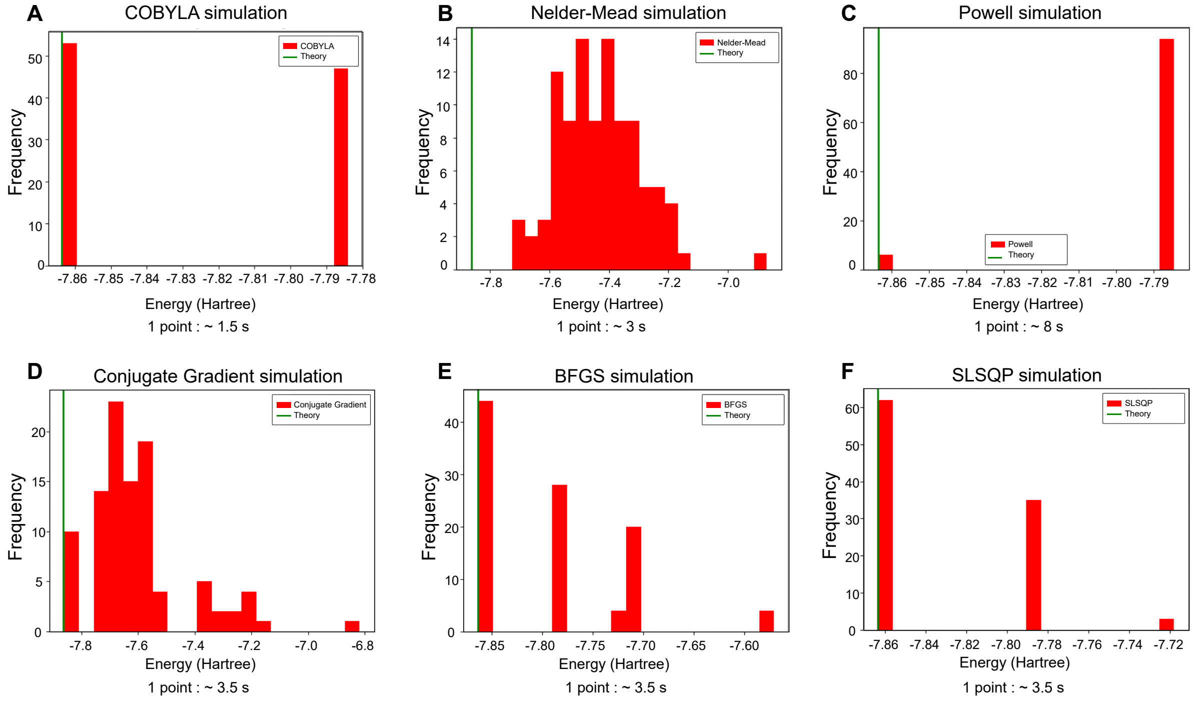

Figure S5. **The results of classical optimizers simulation. A.** COBYLA, **B.** Nelder-Mead, **C.** Powell, **D.** Conjugate gradient, **E.** BFGS, **F.** SLSQP. The green vertical green line corresponds to the theoretical value of the LiH Hamiltonian at a bonding length (1.51 Å) and red bars correspond to the frequency of the converged energy.

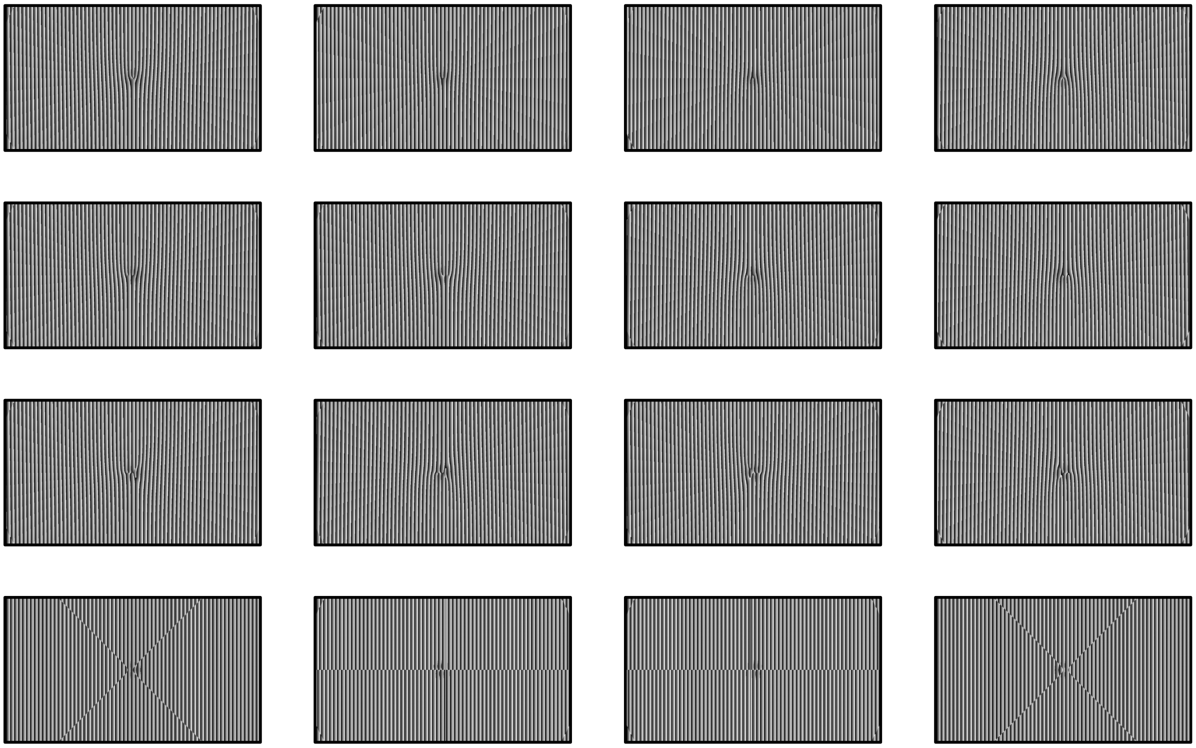

Figure S6. **Pauli Measurement images for He-H+.** These images can be generated using the eigenvectors of the Pauli strings. Each row of images corresponds to a Pauli group. In Group 1, the first row is composed of {II,IZ,ZI,ZZ}; Group 2 includes {IX,ZX}; Group 3 includes {XI,XZ}; and Group 4 includes {XX}.

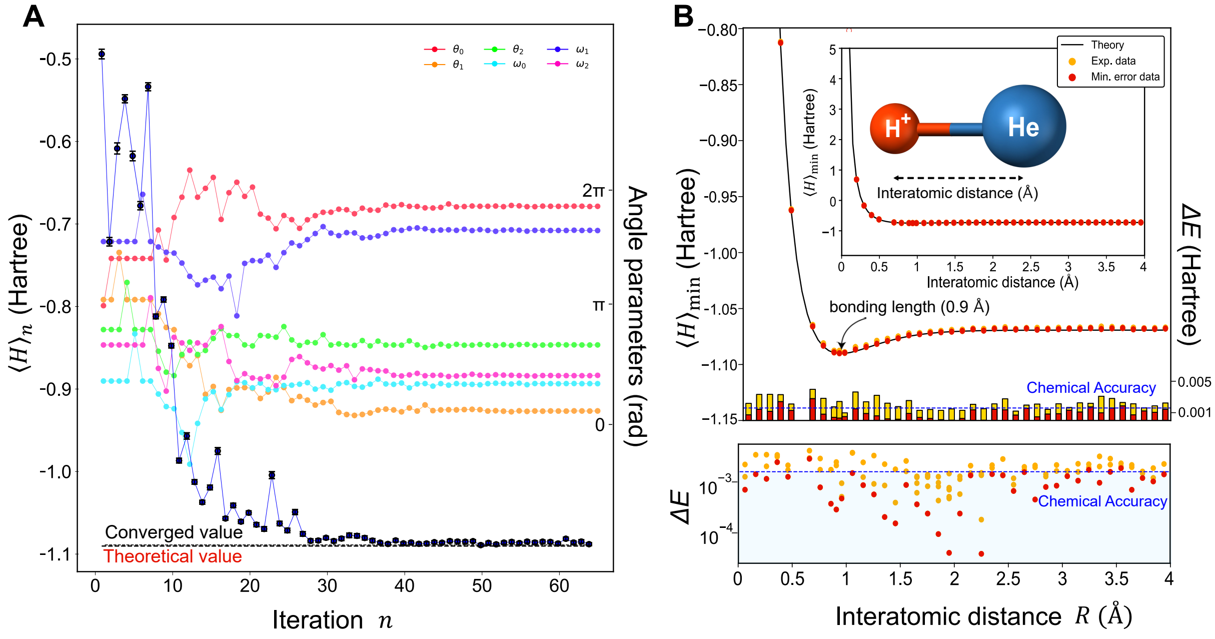

Figure S7. **Experimental results for He–H$^+$. A,** Iterative optimization results of the ground state energy $\langle H \rangle$ of He–H$^+$ at interatomic distance $R = 0.9$ Å, which is the bonding length of He–H$^+$. The blue circles, the red dashed line, the black dashed line correspond to the experimentally estimated ground state energy at each iteration n, the theoretical ground state energy, and the converged ground state energy, respectively. The estimated ground state energy converges to a certain value with 0.0003 Hartree energy difference close to the theoretical value as the number of iterations n increases. The black error bars of the measured energy represent one standard deviation obtained by performing a Monte Carlo simulation 1000 times. (background: Experimental results on 6 angle parameters that control the ansatz qudit state at each iteration. The amount of change on the angle parameters gradually decreases as the number of iterations n increases.) **B,** Estimated ground state energy of He–H$^+$ with respect to the interatomic distance R. ΔE corresponds to the difference between the calculated and the experimentally obtained ground state energies. The blue dotted line corresponds to chemical accuracy. We perform experiment at each interatomic distance multiple times and the red circles correspond to the best result with the minimum energy difference ΔE while the yellow circles correspond to all of the other data. Note that the best data at almost interatomic distance can achieve chemical accuracy, which is approximately 0.0016 Hartree energy difference.

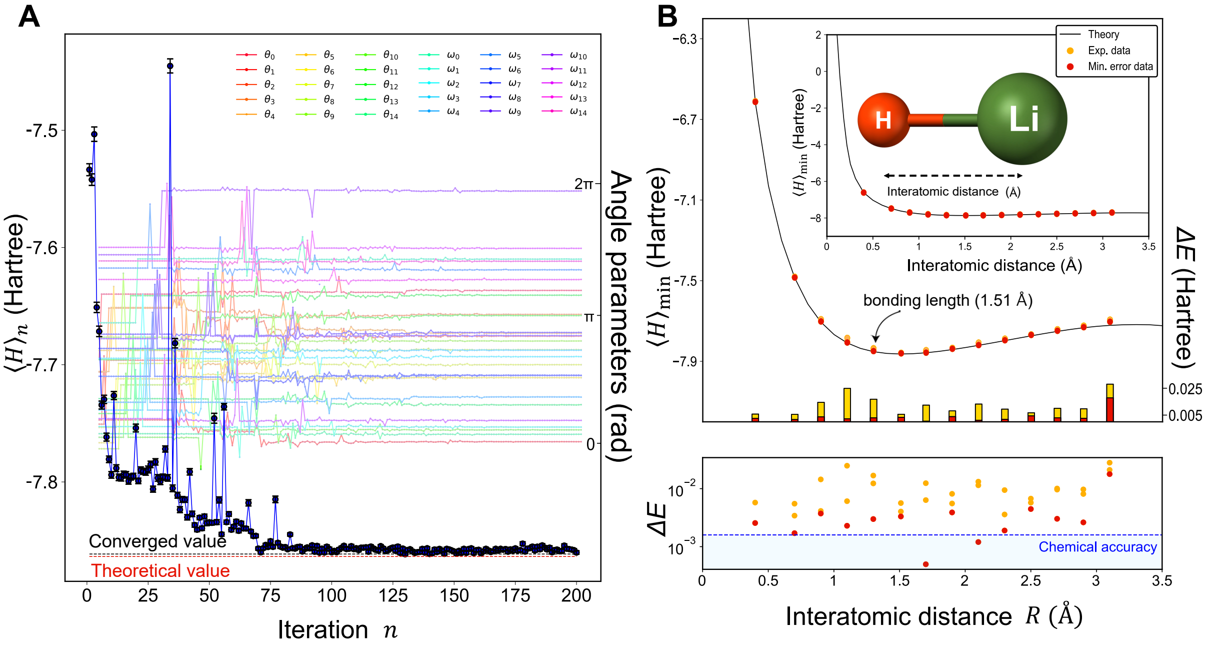

Figure S8. **Experimental results for openfermion-LiH. A,** Iterative optimization results of the ground state energy $\langle H \rangle$ of LiH at interatomic distance $R = 1.51$ Å. The blue circles, the red dashed line, the black dashed line correspond to the experimentally estimated ground state energy at each iteration n, the theoretical ground state energy, and the converged ground state energy, respectively. The estimated ground state energy converges to a certain value with 0.003 Hartree energy difference close to the theoretical value as the number of iterations n increases. The black error bars of the measured energy represent one standard deviation obtained by performing a Monte Carlo simulation 1000 times. (background: Experimental results on 30 angle parameters that control the ansatz qudit state at each iteration. The amount of change on the angle parameters gradually decreases as the number of iterations n increases.) **B,** Estimated ground state energy of LiH with respect to the interatomic distance R. ΔE corresponds to the difference between the calculated and the experimentally obtained ground state energies. The blue dotted line corresponds to chemical accuracy. We perform experiment at each interatomic distance multiple times and the red circles correspond to the best result with the minimum energy difference ΔE while the yellow circles correspond to all of the other data.

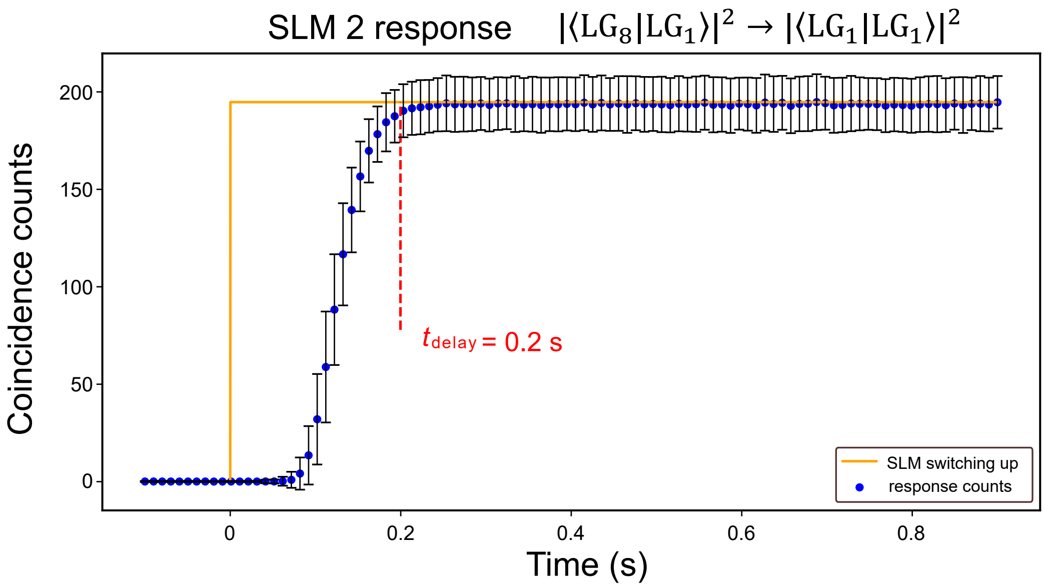

Figure S9. **Result for SLM response time.** SLM1 shows fixed $LG_{l=1}$ image while SLM2 is changed from $LG_{l=8}$ to $LG_{l=1}$. After ∼0.2 s, the coincidence count is stabilized.

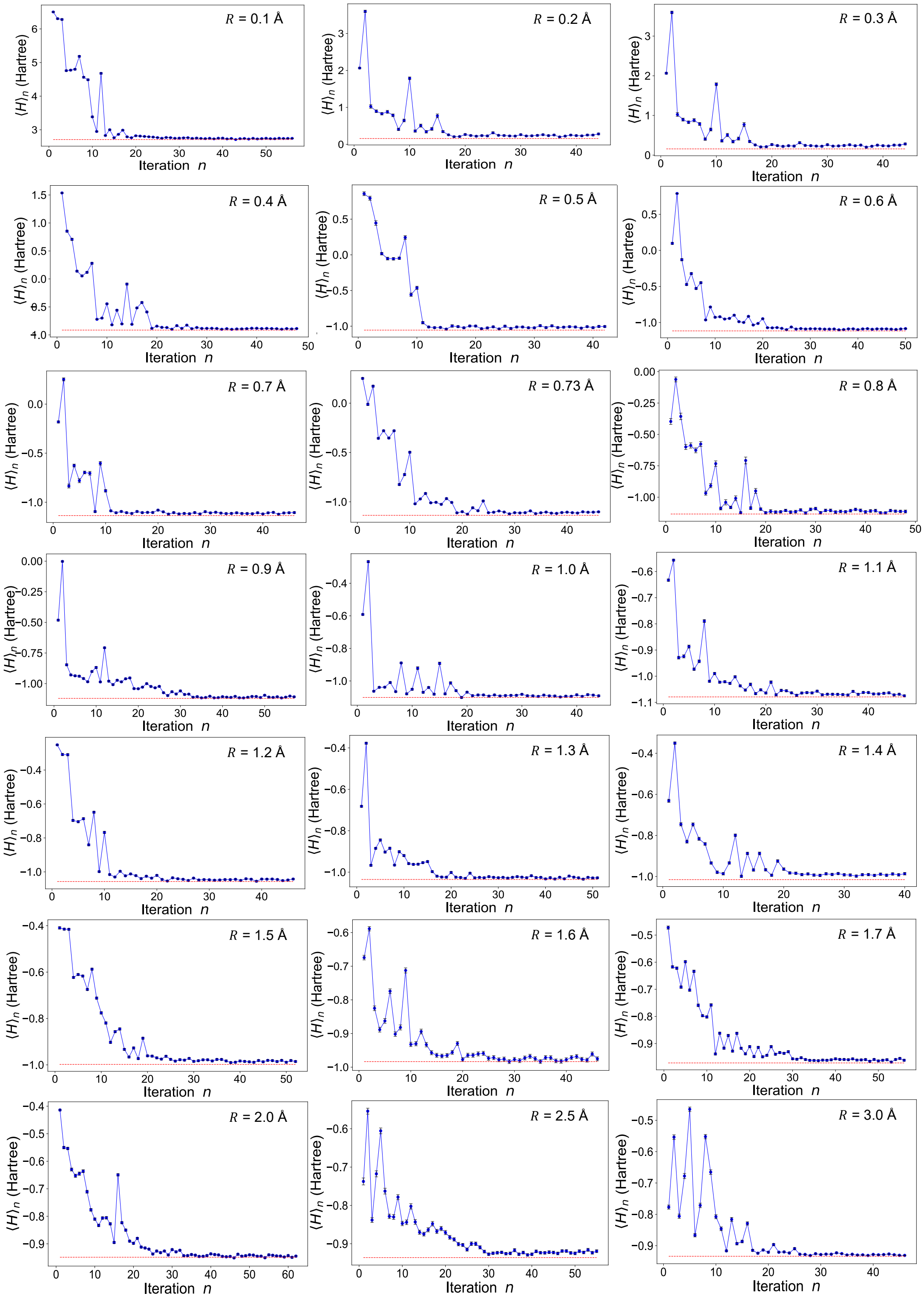

Figure S10. **Iterative optimization results of the ground state energy $\langle H \rangle$ of H_2 at each interatomic distance.**

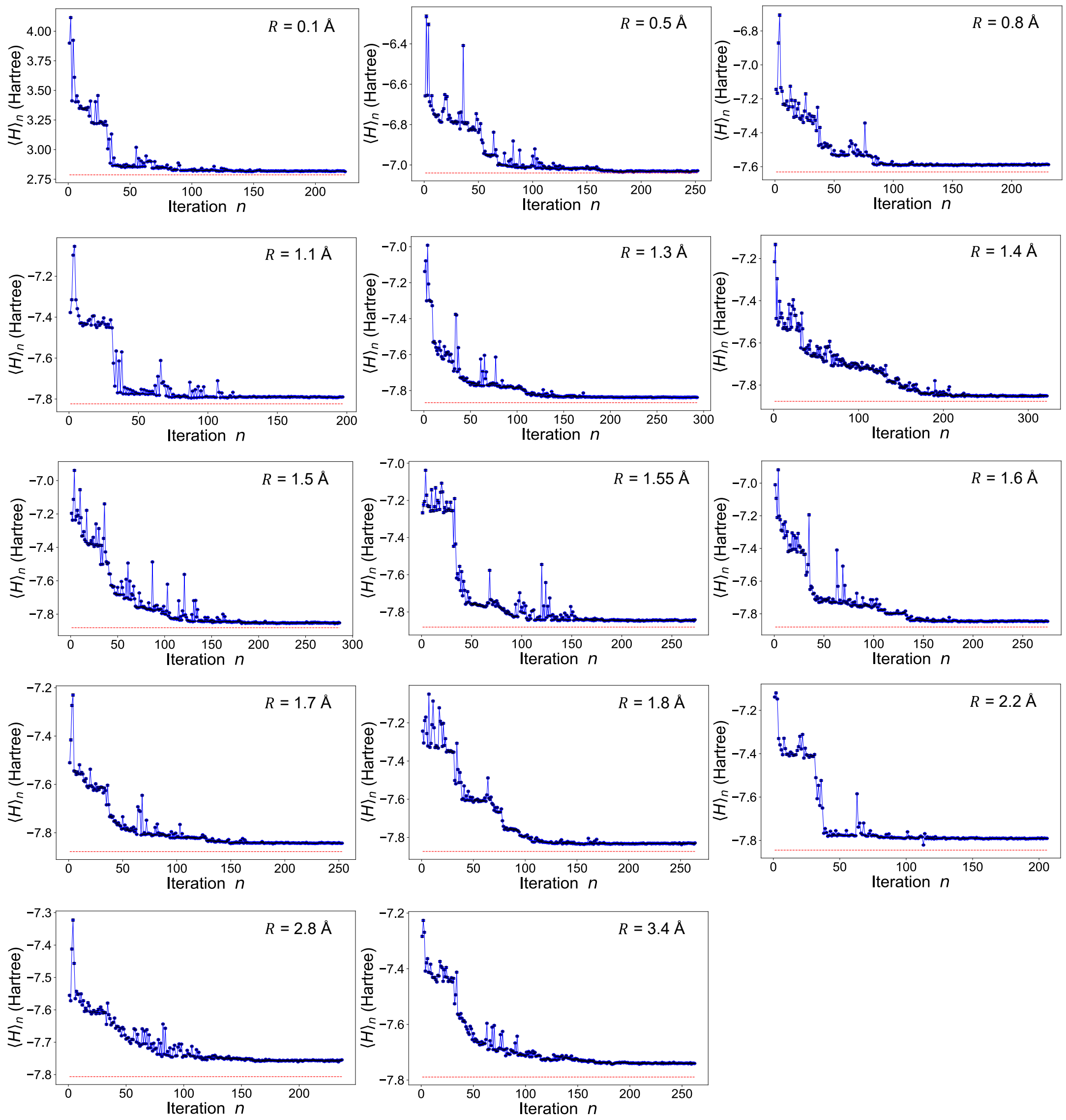

Figure S11. **Iterative optimization results of the ground state energy $\langle H \rangle$ of LiH (Qiskit) at each interatomic distance.**

Captions for Movies S1 to S4

Movie S1. Description for H_2-Qiskit VQE experiment

This video describes the procedure of our experiment, corresponding to Figure 3 in the main text. It explains the construction of the Hamiltonian for the H_2 molecule by Qiskit library. molecule using the Qiskit library. Color and intensity are employed to illustrate how the ansatz state is mapped to the LG mode in each iteration. Finally, the video shows the evolution of the ansatz and the convergence of the eigenvalue based on our experimental data.

Movie S2. Description for LiH-Qiskit VQE experiment

This video describes the procedure of our experiment, corresponding to Figure 4 in the main text. It explains the construction of the Hamiltonian for the LiH molecule by Qiskit library. molecule using the Qiskit library. Color and intensity are employed to illustrate how the ansatz state is mapped to the LG mode in each iteration. Finally, the video shows the evolution of the ansatz and the convergence of the eigenvalue based on our experimental data.

Movie S3. Description for He-H^+-Openfermion VQE experiment

This video describes the procedure of our experiment, corresponding to Figure S7. It explains the construction of the Hamiltonian for the He-H^+ molecule using Openfermion library. Color and intensity are employed to illustrate how the ansatz state is mapped to the LG mode in each iteration. Finally, the video shows the evolution of the ansatz and the convergence of the eigenvalue based on our experimental data.

Movie S4. Description for LiH-Openfermion VQE experiment

This video describes the procedure of our experiment, corresponding to Figure S8. It explains

the construction of the Hamiltonian for the LiH molecule by Openfermion library. Color and intensity are employed to illustrate how the ansatz state is mapped to the LG mode in each iteration. Finally, the video shows the evolution of the ansatz and the convergence of the eigenvalue based on our experimental data.

REFERENCES AND NOTES

1. M. Cerezo, A. Arrasmith, R. Babbush, S. C. Benjamin, S. Endo, K. Fujii, J. R. McClean, K. Mitarai, X. Yuan, L. Cincio, P. J. Coles, Variational quantum algorithms. *Nat. Rev. Phys.* **3**, 625–644 (2021).

2. J. Preskill, Quantum computing in the NISQ era and beyond. *Quantum* **2**, 79 (2018).

3. J. Tilly, H. Chen, S. Cao, D. Picozzi, K. Setia, Y. Li, E. Grant, L. Wossnig, I. Rungger, G. H. Booth, J. Tennyson, The variational quantum eigensolver: A review of methods and best practices. *Phys. Rep.* **986**, 1–128 (2022).

4. A. Peruzzo, J. McClean, P. Shadbolt, M.-H. Yung, X.-Q. Zhou, P. J. Love, A. Aspuru-Guzik, J. L. O'Brien, A variational eigenvalue solver on a photonic quantum processor. *Nat. Commun.* **5**, 4213 (2014).

5. D. Lee, J. Lee, S. Hong, H.-T. Lim, Y.-W. Cho, S.-W. Han, H. Shin, J. ur Rehman, Y.-S. Kim, Error-mitigated photonic variational quantum eigensolver using a single-photon ququart. *Optica* **9**, 88–95 (2022).

6. P. J. J. O'Malley, R. Babbush, I. D. Kivlichan, J. Romero, J. R. McClean, R. Barends, J. Kelly, P. Roushan, A. Tranter, N. Ding, B. Campbell, Y. Chen, Z. Chen, B. Chiaro, A. Dunsworth, A. G. Fowler, E. Jeffrey, E. Lucero, A. Megrant, J. Y. Mutus, M. Neeley, C. Neill, C. Quintana, D. Sank, A. Vainsencher, J. Wenner, T. C. White, P. V. Coveney, P. J. Love, H. Neven, A. Aspuru-Guzik, J. M. Martinis, Scalable quantum simulation of molecular energies. *Phys. Rev. X* **6**, 031007 (2016).

7. F. Arute, K. Arya, R. Babbush, D. Bacon, J. C. Bardin, R. Barends, S. Boixo, M. Broughton, B. B. Buckley, D. A. Buell, B. Burkett, N. Bushnell, Y. Chen, Z. Chen, B. Chiaro, R. Collins, W. Courtney, S. Demura, A. Dunsworth, E. Farhi, A. Fowler, B. Foxen, C. Gidney, M. Giustina, R. Graff, S. Habegger, M. P. Harrigan, A. Ho, S. Hong, T. Huang, W. J. Huggins, L. Ioffe, S. V. Isakov, E. Jeffrey, Z. Jiang, C. Jones, D. Kafri, K. Kechedzhi, J. Kelly, S. Kim, P. V. Klimov, A. Korotkov, F. Kostritsa, D. Landhuis, P. Laptev, M. Lindmark, E. Lucero, O. Martin, J. M. Martinis, J. R. McClean, M. McEwen, A. Megrant, X. Mi, M. Mohseni, W. Mruczkiewicz, J. Mutus, O. Naaman, M. Neeley, C. Neill, H. Neven, M. Y. Niu, T. E. O'Brien,

E. Ostby, A. Petukhov, H. Putterman, C. Quintana, P. Roushan, N. C. Rubin, D. Sank, K. J. Satzinger, V. Smelyanskiy, D. Strain, K. J. Sung, M. Szalay, T. Y. Takeshita, A. Vainsencher, T. White, N. Wiebe, Z. J. Yao, P. Yeh, A. Zalcman, Hartree-fock on a superconducting qubit quantum computer. *Science* **369**, 1084–1089 (2020).

8. A. Kandala, A. Mezzacapo, K. Temme, M. Takita, M. Brink, J. M. Chow, J. M. Gambetta, Hardware-efficient variational quantum eigensolver for small molecules and quantum magnets. *Nature* **549**, 242–246 (2017).

9. A. Kandala, K. Temme, A. D. Córcoles, A. Mezzacapo, J. M. Chow, J. M. Gambetta, Error mitigation extends the computational reach of a noisy quantum processor. *Nature* **567**, 491–495 (2019).

10. C. Hempel, C. Maier, J. Romero, J. McClean, T. Monz, H. Shen, P. Jurcevic, B. P. Lanyon, P. Love, R. Babbush, A. Aspuru-Guzik, R. Blatt, C. F. Roos, Quantum chemistry calculations on a trapped-ion quantum simulator. *Phys. Rev. X* **8**, 031022 (2018).

11. Y. Wang, Z. Hu, B. Sanders, S. Kais, Qudits and high-dimensional quantum computing. *Front. Phys.* **8**, 589504 (2020).

12. D. Cozzolino, B. Da Lio, D. Bacco, L. K. Oxenløwe, High-dimensional quantum communication: Benefits, progress, and future challenges. *Adv. Quantum Technol.* **2**, 1900038 (2019).

13. M. Luo, X. Wang, Universal quantum computation with qudits. *Sci. China Phys. Mech. Astron.* **57**, 1712–1717 (2014).

14. P. Liu, R. Wang, J.-N. Zhang, Y. Zhang, X. Cai, H. Xu, Z. Li, J. Han, X. Li, G. Xue, W. Liu, L. You, Y. Jin, H. Yu, Performing SU(d) operations and rudimentary algorithms in a superconducting transmon qudit for $d = 3$ and $d = 4$. *Phys. Rev. X* **13**, 021028 (2023).

15. S. Cao, M. Bakr, G. Campanaro, S. D. Fasciati, J. Wills, D. Lall, B. Shteynas, V. Chidambaram, I. Rungger, P. Leek, Emulating two qubits with a fourlevel transmon qudit for variational quantum algorithms. *Quantum Sci. Technol.* **9**, 035003 (2024).

16. M. Ringbauer, M. Meth, L. Postler, R. Stricker, R. Blatt, P. Schindler, T. Monz, A universal qudit quantum processor with trapped ions. *Nat. Phys.* **18**, 1053–1057 (2022).

17. P. Hrmo, B. Wilhelm, L. Gerster, M. W. van Mourik, M. Huber, R. Blatt, P. Schindler, T. Monz, M. Ringbauer, Native qudit entanglement in a trapped ion quantum processor. *Nat. Commun.* **14**, 2242 (2023).

18. A. M. Yao, M. J. Padgett, Orbital angular momentum: Origins, behavior and applications. *Adv. Opt. Photonics* **3**, 161–204 (2011).

19. M. Rambach, M. Qaryan, M. Kewming, C. Ferrie, A. G. White, J. Romero, Robust and efficient high-dimensional quantum state tomography. *Phys. Rev. Lett.* **126**, 100402 (2021).

20. R. Chen, H. Zhou, M. Moretti, X. Wang, J. Li, Orbital angular momentum waves: Generation, detection, and emerging applications. *IEEE Commun. Surv. Tutor.* **22**, 840–868 (2020).

21. E. Bolduc, N. Bent, E. Santamato, E. Karimi, R. W. Boyd, Exact solution to simultaneous intensity and phase encryption with a single phase-only hologram. *Opt. Lett.* **38**, 3546–3549 (2013).

22. F. Brandt, M. Hiekkamäki, F. Bouchard, M. Huber, R. Fickler, High-dimensional quantum gates using full-field spatial modes of photons. *Optica* **7**, 98–107 (2020).

23. M. Mirhosseini, O. S. Magaña-Loaiza, M. N. O'Sullivan, B. Rodenburg, M. Malik, M. P. J. Lavery, M. J. Padgett, D. J. Gauthier, R. W. Boyd, High-dimensional quantum cryptography with twisted light. *New J. Phys.* **17**, 033033 (2015).

24. M. Hiekkamäki, F. Bouchard, R. Fickler, Photonic angular superresolution using twisted N00N states. *Phys. Rev. Lett.* **127**, 263601 (2021).

25. M. Born, R. Oppenheimer, On the quantum theory of molecules, in *Quantum Chemistry: Classic Scienti ic Papers* (World Scientific, 2000), pp. 1–24.

26. T. D. Schultz, D. C. Mattis, E. H. Lieb, Two-dimensional ising model as a soluble problem of many fermions. *Rev. Mod. Phys.* **36**, 856–871 (1964).

27. F. C. R. Peres, Pauli-based model of quantum computation with higher-dimensional systems. *Phys. Rev. A* **108**, 032606 (2023).

28. F. Schlederer, M. Krenn, R. Fickler, M. Malik, A. Zeilinger, Cyclic transformation of orbital angular momentum modes. *New J. Phys.* **18**, 043019 (2016).

29. A. Babazadeh, M. Erhard, F. Wang, M. Malik, R. Nouroozi, M. Krenn, A. Zeilinger, High-dimensional single-photon quantum gates: Concepts and experiments. *Phys. Rev. Lett.* **119**, 180510 (2017).

30. X. Gao, M. Krenn, J. Kysela, A. Zeilinger, Arbitrary d-dimensional pauli X gates of a flying qudit. *Phys. Rev. A* **99**, 023825 (2019).

31. N. Bent, H. Qassim, A. A. Tahir, D. Sych, G. Leuchs, L. L. Sánchez-Soto, E. Karimi, R. W. Boyd, Experimental realization of quantum tomography of photonic qudits via symmetric informationally complete positive operator-valued measures. *Phys. Rev. X* **5**, 041006 (2015).

32. H. Qassim, F. M. Miatto, J. P. Torres, M. J. Padgett, E. Karimi, R. W. Boyd, Limitations to the determination of a Laguerre-Gauss spectrum via projective, phase-flattening measurement. *J. Opt. Soc. Am. B* **31**, A20–A23 (2014).

33. O. Lockwood, Paper Reviews (2022, GitHub); https://github.com/lockwo/Paper-Review/tree/main/HEA-VQE.

34. A. Forbes, A. Dudley, M. McLaren, Creation and detection of optical modes with spatial light modulators. *Adv. Opt. Photonics* **8**, 200–227 (2016).

35. A. E. Willner, H. Huang, Y. Yan, Y. Ren, N. Ahmed, G. Xie, C. Bao, L. Li, Y. Cao, Z. Zhao, J. Wang, M. P. J. Lavery, M. Tur, S. Ramachandran, A. F. Molisch, N. Ashrafi, S. Ashrafi, Optical communications using orbital angular momentum beams. *Adv. Opt. Photonics* **7**, 66–106 (2015).

36. X. Yan, P. F. Zhang, J. H. Zhang, H. Q. Chun, C. Y. Fan, Decoherence of orbital angular momentum tangled photons in non-kolmogorov turbulence. *J. Opt. Soc. Am. A Opt. Image Sci. Vis.* **33**, 1831–1835 (2016).

37. M. Erhard, R. Fickler, M. Krenn, A. Zeilinger, Twisted photons: New quantum perspectives in high dimensions. *Light Sci. Appl.* **7**, 17146 (2018).

38. E. Bolduc, G. Gariepy, J. Leach, Direct measurement of large-scale quantum states via expectation values of non-hermitian matrices. *Nat. Commun.* **7**, 10439 (2016).

39. I. Nape, V. Rodriguez-Fajardo, F. Zhu, H.-C. Huang, J. Leach, A. Forbes, Measuring dimensionality and purity of high-dimensional entangled states. *Nat. Commun.* **12**, 5159 (2021).

40. J. Bavaresco, N. H. Valencia, C. Klöckl, M. Pivoluska, P. Erker, N. Friis, M. Malik, M. Huber, Measurements in two bases are sufficient for certifying high-dimensional entanglement. *Nat. Phys.* **14**, 1032–1037 (2018).

41. M. Krenn, M. Huber, R. Fickler, R. Lapkiewicz, S. Ramelow, A. Zeilinger, Generation and confirmation of a (100 × 100)-dimensional entangled quantum system. *Proc. Natl. Acad. Sci. U.S.A.* **111**, 6243–6247 (2014).

42. Y. Chi, J. Huang, Z. Zhang, J. Mao, Z. Zhou, X. Chen, C. Zhai, J. Bao, T. Dai, H. Yuan, M. Zhang, D. Dai, B. Tang, Y. Yang, Z. Li, Y. Ding, L. K. Oxenløwe, M. G. Thompson, J. L. O'Brien, Y. Li, Q. Gong, J. Wang, A programmable qudit-based quantum processor. *Nat. Commun.* **13**, 1166 (2022).

43. Y. Wang, S. Ru, F. Wang, P. Zhang, F. Li, Experimental demonstration of efficient high-dimensional quantum gates with orbital angular momentum. *Quantum Sci. Technol.* **7**, 015016 (2022).

44. A. C. Dada, J. Leach, G. S. Buller, M. J. Padgett, M. J. E. Andersson, Experimental high-dimensional two photon entanglement and violations of generalized bell inequalities. *Nat. Phys.* **7**, 677–680 (2011).

45. M. Erhard, M. Malik, M. Krenn, A. Zeilinger, Experimental Greenberger-Horne-Zeilinger entanglement beyond qubits. *Nat. Photonics* **12**, 759–764 (2018).

46. M. Malik, M. Erhard, M. Huber, M. Krenn, R. Fickler, A. Zeilinger, Multi-photon entanglement in high dimensions. *Nat. Photonics* **10**, 248–252 (2016).

47. J. T. Barreiro, T. C. Wei, P. G. Kwiat, Remote preparation of single-photon "hybrid" entangled and vector-polarization states. *Phys. Rev. Lett.* **105**, 030407 (2010).

48. F. Kaneda, P. G. Kwiat, High-efficiency single-photon generation via large-scale active time multiplexing. *Sci. Adv.* **5**, eaaw8586 (2019).

49. Y. Cao, J. Romero, A. Aspuru-Guzik, Potential of quantum computing for drug discovery. *IBM J. Res. Dev.* **62**, 6:1–6:20 (2018).

50. P. J. Low, B. M. White, A. A. Cox, M. L. Day, C. Senko, Practical trapped-ion protocols for universal qudit-based quantum computing. *Phys. Rev. Res.* **2**, 033128 (2020).

51. A. Fedrizzi, T. Herbst, A. Poppe, T. Jennewein, A. Zeilinger, A wavelength-tunable fiber-coupled source of narrowband entangled photons. *Opt. Express* **15**, 15377–15386 (2007).

52. W. Shao, S. Huang, M. Chen, X. Liu, W. Xie, Research of optical vortex's energy efficiency and diffraction angle based on spatial light modulator. *Opt. Eng.* **56**, 086113 (2017).

53. Y. Herasymenko, T. E. O'Brien, A diagrammatic approach to variational quantum ansatz construction. *Quantum* **5**, 596 (2021).

54. C. Rosales-Guzmán, A. Forbes, "Generation Hologram," *In How to shape light with spatial light modulators* (SPIE, 2017), pp. 1–57.

55. J. R. McClean, N. C. Rubin, K. J. Sung, I. D. Kivlichan, X. Bonet-Monroig, Y. Cao, C. Dai, E. S. Fried, C. Gidney, B. Gimby, P. Gokhale, T. Häner, T. Hardikar, V. Havlíček, O. Higgott, C. Huang, J. Izaac, Z. Jiang, X. Liu, S. M. Ardle, M. Neeley, T. O'Brien, B. O'Gorman, I. Ozfidan, M. D. Radin, J. Romero, N. P. D. Sawaya, B. Senjean, K. Setia, S. Sim, D. S.

Steiger, M. Steudtner, Q. Sun, W. Sun, D. Wang, F. Zhang, R. Babbush, OpenFermion: The electronic structure package for quantum computers. *Quantum Sci. Technol.* **5**, 034014 (2020).

56. V. Bergholm, J. Izaac, M. Schuld, C. Gogolin, S. Ahmed, V. Ajith, M. S. Alam, G. Alonso-Linaje, B. AkashNarayanan, A. Asadi, J. M. Arrazola, U. Azad, S. Banning, C. Blank, T. R. Bromley, B. A. Cordier, J. Ceroni, A. Delgado, O. D. Matteo, A. Dusko, T. Garg, D. Guala, A. Hayes, R. Hill, A. Ljaz, T. Isacsson, D. Ittah, S. Jahangiri, P. Jain, E. Jiang, A. Khandelwal, K. Kottmann, R. A. Lang, C. Lee, T. Loke, A. Lowe, K. McKiernan, J. Jakob M., J. A. Montañez-Barrera, R. M., Z. Niu, L. J. O'Riordan, S. Oud, A. Panigrahi, C.-Y. Park, D. Polatajko, N. Quesada, C. Roberts, N. Sá, I. Schoch, B. Shi, S. Shu, S. Sim, A. Singh, I. Strandberg, J. Soni, A. Száva, S. Thabet, R. A. Vargas-Hernández, T. Vincent, N. Vitucci, M. Weber, D. Wierichs, R. Wiersema, M. Willmann, V. Wong, S. Zhang, N. Killoran, PennyLane: Automatic differentiation of hybrid quantum-classical computations. arXiv:1811.04968 [quant-ph] (2022).

57. M. J. D. Powell, On trust region methods for unconstrained minimization without derivatives. *Math. Program.* **97**, 605–623 (2003).
